# The blood–brain barrier: Structure, regulation and drug delivery

**DOI:** 10.1038/s41392-023-01481-w

**Published:** 2023-05-25

**Authors:** Di Wu, Qi Chen, Xiaojie Chen, Feng Han, Zhong Chen, Yi Wang

**Affiliations:** 1 Key Laboratory of Neuropharmacology and Translational Medicine of Zhejiang Province, School of Pharmaceutical Sciences, Zhejiang Chinese Medical University, 310053 Hangzhou, China; 2 Zhejiang Rehabilitation Medical Center, The Third Affiliated Hospital of Zhejiang Chinese Medical University, 310053 Hangzhou, China; 3 Key Laboratory of Cardiovascular & Cerebrovascular Medicine, Drug Target and Drug Discovery Center, School of Pharmacy, Nanjing Medical University, Nanjing, China

**Keywords:** Drug delivery, Neurological disorders

## Abstract

Blood–brain barrier (BBB) is a natural protective membrane that prevents central nervous system (CNS) from toxins and pathogens in blood. However, the presence of BBB complicates the pharmacotherapy for CNS disorders as the most chemical drugs and biopharmaceuticals have been impeded to enter the brain. Insufficient drug delivery into the brain leads to low therapeutic efficacy as well as aggravated side effects due to the accumulation in other organs and tissues. Recent breakthrough in materials science and nanotechnology provides a library of advanced materials with customized structure and property serving as a powerful toolkit for targeted drug delivery. In-depth research in the field of anatomical and pathological study on brain and BBB further facilitates the development of brain-targeted strategies for enhanced BBB crossing. In this review, the physiological structure and different cells contributing to this barrier are summarized. Various emerging strategies for permeability regulation and BBB crossing including passive transcytosis, intranasal administration, ligands conjugation, membrane coating, stimuli-triggered BBB disruption, and other strategies to overcome BBB obstacle are highlighted. Versatile drug delivery systems ranging from organic, inorganic, and biologics-derived materials with their synthesis procedures and unique physio-chemical properties are summarized and analyzed. This review aims to provide an up-to-date and comprehensive guideline for researchers in diverse fields, offering perspectives on further development of brain-targeted drug delivery system.

## Introduction

Blood–brain barrier (BBB) is a semi-permeable barrier encompassing microvasculature of central nervous system (CNS). In the capillaries, the wedged endothelial cells line in the interior vessels forming extensive tight junctions.^1–3^ Together with an ensemble of receptors, transporters, efflux pumps and other cellular components, the barrier takes control of entrance and expulsion of the molecules in vascular compartment to the brain. The intact BBB impedes the influx of most blood-borne substances from entering the brain. But it should be noted that at meantime of brain protection, BBB also excludes more than 98% of small-molecule drugs and all macromolecular therapeutics from access to the brain.^4,5^ The tight gap allows only passive diffusion of lipid-soluble drugs at a molecular weight lower than 400-600 Da. Increasing lipophilicity of the therapeutic agents is a feasible method to improve the BBB permeability. For example, Crizotinib, an oral selective small-molecular tyrosine kinase inhibitor, is an effective anti-cancer medicine but with poor activity against brain tumor metastases due to its low BBB penetration.^6^ Structural modification of conjugation of a fluoroethyl moiety increases the lipophilicity of Crizotinib and results in enhanced brain permeability.^7^ However, increasing lipophilicity is not a universal strategy as it may inhibit the biological activity of drugs of interest. Further, therapeutic drugs with high liposolubility have longer retention and duration of action in non-target peripheral organs, causing considerable side effects. In addition, due to the presence of P-glycoprotein (referred to as multidrug resistance associated membrane protein), drugs could be transported back into the blood by ATP-dependent efflux pumps.^8,9^ Thus, it is urging to address the issue of brain-targeted therapeutics by developing effective and safe delivery strategies.

**Fig. 1:**
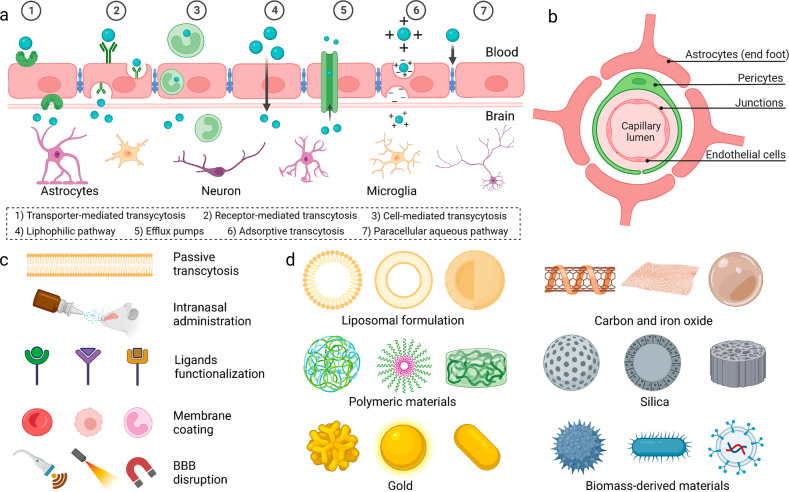
Strategies and materials for BBB regulation and brain-targeted drug delivery. **a** Schematic diagram of different mechanisms for BBB crossing. **b** Schematic diagram of BBB structure. **c** Engineered materials for brain-targeted drug delivery. **d** Various non-invasive strategies for BBB crossing

Spurred by recent development of materials science and nanotechnology, various strategies for regulation of BBB permeability were developed as well as a library of brain-targeted drug delivery systems. Transport routes of the drug molecules across the BBB occurs via the pathways including paracellular and transcellular diffusion, receptor-mediated transcytosis, cell-mediated transcytosis, transporter-mediated transcytosis, and adsorptive mediated transcytosis (Fig. 1a).^10^ Many efforts have been made in response to each process of the drug transportation, which have also been accompanied by reviews of latest progress in this field, but most of literature emphasize the BBB breakdown in specific brain disorders,^11–14^ or give a general review of delivery strategies concentrating on the barrier physiology.^15,16^ On the basis of these strategies, functional materials with small size, tailored architecture and therapeutic motifs that facilitate the targeted drug delivery are widely engineered. They can be constructed by using a wide range of substrates including organic (e.g., liposomes, micelles, hydrogels, etc.),^17–19^ inorganic (e.g., metal/metal oxide particles, silica nanoparticles, quantum dots, etc.),^20,21^ and biomass-derived materials (e.g., exosomes, cells, bacteria, etc.).^22–24^ Improved performance of brain-targeted drug delivery was proved thanks to these materials with obvious advantages including non-invasive delivery, high drug loading, good biocompatibility, prolonged blood circulation, and importantly brain targeting effect. Over the past few years, we have witnessed the booming advances in exploiting materials science and nanotechnology in brain-targeted drug delivery. Brain disorder therapy utilizing tailored systems has been a benefactor of this emergence, yet a timely and comprehensive review is lacking. In the light of excellent performance of BBB crossing strategies for enhanced drug delivery, we will first summarize the structure and different cells contributing to the integrity of BBB (Fig. 1b). Further, main BBB crossing strategies derived are highlighted with relevant transcytosis mechanisms as well as their interactions at barrier interface (Fig. 1c). Specifically, we addressed the issues of different pathways from passive transcytosis, intranasal administration, ligands conjugation for brain targeting, membrane coating for brain targeting, to stimuli-mediated BBB disruption. Then, the updated progress of customized materials was overviewed and how they were fabricated and utilized for the treatment of brain disorders was summarized (Fig. 1d). The discussion of potential translational trials and the remaining challenging in this field will also be presented. This review will be of great interest to those working in materials science, nanotechnology, and especially to those in biomedical engineering and translational medicine, which will not only serve as an up-to-date compilation of achievements in this area, but also provide a generalized guideline for BBB regulation and rational design of targeted drug delivery systems.

## BBB structure and physiology

### Anatomical structure of the BBB

The existence of the BBB was first found by Paul Ehrlich and proved by Edwin Goldmann.^25,26^ BBB is a spacious, multicellular, and dynamic semi-permeable membrane that isolates the foreign substances in the blood from the CNS.^27^ The presence of BBB avoids the brain from damage by keeping a stable environment.^28,29^ But it also limits the drugs that enter into the CNS for treating brain diseases such as neurodegenerative diseases and brain cancer.^30,31^ Capillaries are the major site of the BBB. Because the neural cell is close to a capillary no further than 25 µm, passaging the BBB for drug delivery is a favored route when compared to another by-passed route which is relatively longer.^32^ This case pushes researchers to develop effective strategies to regulate BBB permeability and targeted delivery systems to overcome the BBB limits. Some reviews have discussed the BBB recognition which is one of the key steps for enhanced brain-targeted delivery.^33–35^ For a deeper understating of the interaction between delivery systems and the brain, how the BBB is constructed should be clarified.

From the physiological point of view, endothelial cells, astroglia, pericytes, and junctional complexes including tight junctions and adherens junctions compose the BBB basically.^36–39^ In this section, we focus on the five constituents mentioned above and others are not covered here.

#### Endothelial cells

Endothelial cells are considered as the BBB’s core anatomical structure for lining the cerebral blood vessels and interacting with different types of cells in the CNS.^40,41^ The endothelial cells in the BBB differ from peripheral endothelial cells in morphology and function.^42,43^ The barrier performance is not the innate properties of endothelial cells.^44,45^ For morphology, the endothelial cells in the BBB are fastened by both tight junctions and adherens junctions, resulting in distinct lumenal and abluminal membrane compartments.^46^ They further present with no fenestrations, also known as small transcellular pores, which greatly limit free diffusion and the rapid exchange of molecules between brain tissue and blood.^47^ Besides, the amounts of mitochondria in endothelial cells in the BBB are higher than in peripheral endothelial cells, which means more energy is needed for transport.^48,49^ For function, first, they display a net negative surface charge, refusing to accept negatively charged compounds, as well as quite low degrees of leukocyte adhesion molecules, hampering the entry of the number of immune cells.^50–52^ Second, they show designated transporters for regulating the inflow and outflow of specific substrates.^53^ Third, they show a restriction on the number of transcellular vesicles through the vessel wall due to the high transendothelial electrical resistance.^54,55^ Because of the existence of the local environment, endothelial cells can together form and maintain the BBB.

#### Astrocytes

Astrocytes, also known as astroglia, are the most numerous glial cells, expressing polarized and complex morphology, which are heterogeneous throughout the brain.^56^ In tradition, they are divided into two categories, that one is protoplasmic which locates in the well-vascularized gray matter, and the other one is fibrous which locates in the less vascular white matter.^57–59^ Their end feet link them with the basement membrane, via the binding of a set of proteins (aquaporin IV and the dystroglycan-dystrophin complex) with the proteoglycan agrin.^60,61^ In the CNS, they play a major role in dynamic signaling such as clearing waste, tuning brain blood flow, regulating vascular function, ion hemostasis and balancing neuroimmune responses.^62–66^ However, the exact role of astrocytes in BBB function is still controversial.^67–69^ Some studies considered astrocytes can arise barrier behaviors in cerebral, other endothelial cells, and related epithelial, while other studies thought the BBB goes first before the appearance of astrocytes. In this regard, there is no doubt that the BBB exists primarily through the coordination between cells, and astrocytes are a type of neural cells that together with pericytes surround blood vessels in the brain, serving as the interface between neurons and endothelial cells.^70,71^ Moreover, for invertebrates that lack a vascularized circulatory system, astrocytes are the main components of the barrier separating humoral fluids from the CNS.^72^

#### Pericytes

Pericytes are mural cells presenting at intervals along the walls of the capillary blood vessels.^73^ They are embedded in the basement membrane and lie abluminal to the endothelial cells.^74^ The length that pericytes covering the CNS endothelium approaches 100%.^75^ It should be noted that pericytes are central to the neurovascular unit function.^76–78^ Because of the physical apposition, pericytes and endothelial cells are in close communicate with each other. For example, the PDGF-B signaling pathway is one such communication.^79,80^ Endothelial cells secrete the PDGF-B to bind PDGFRβ on pericytes, which recruits pericytes to blood vessels.^81,82^ In turn, pericytes also can release signaling factors to affect endothelial cells by determining the numbers of tight junctions and polarizing the end feet of astrocytes.^83^ If the amounts of pericytes reduce, the tight junctions between endothelial cells will also be reduced.^84^ Except for modulating and maintaining the BBB, pericytes also have functions in adjusting cerebral blood flow, vascular development and maintenance, and neuroinflammation.^85–87^

#### Tight junctions

In the BBB, tight junctions are the main functional components in sustaining the permeability barrier and controlling tissue homeostasis.^88–91^ They are also known as occluding junctions or zonulae occludes which can restrict the cross of hydrophilic molecules and macromolecules.^92^ They reside between endothelial cells, seal the interendothelial cleft, and work as gates and fences to limit paracellular permeability and the lateral diffusion of integral membrane proteins and lipids, thus maintaining cell polarization.^93,94^ There are many transmembrane and cytoplasmic proteins involved in forming tight junctions. Claudins and occludins that situate in two-cell contacts are the major tight junction proteins.^95,96^ Claudins display essential barrier function and occludins ensure the tightness of the tight junctions.^97^ Tricellulin and the lipolysis-stimulated lipoprotein receptor that reside in three-cell contacts are also the major tight junction proteins.^98,99^ Besides, more like junction adhesion molecules, calcium/calmodulin-dependent serine protein kinase, monoclonal antibody 7H6, and heterotrimeric G protein also contribute to the constitution of tight junctions.^100–103^ Junction adhesion molecules mediate the early attachment in the BBB developmental processes.^104,105^ Kinase-like proteins modulate the blood–brain barrier permeability.^106^ Interactions among these proteins provide physical support for the complex of tight junctions.^107^ Downregulation of tight junction-related proteins will loss of the BBB phenotype.^108^

#### Adherens junctions

Adherens junction is extra crucial for the BBB structural integrity and appropriate assembly of proteins of tight junctions.^109,110^ At cellular interfaces, they build spatially, chemically, and mechanically discrete microdomains.^111^ In common with tight junctions, adherens junctions are linked to the cytoskeleton and consisted of transmembrane and cytoplasmic plaque proteins.^112,113^ Vascular endothelial-cadherin, scaffolding proteins catenins, scaffolding proteins p120, plakoprotein are the main components of adherens junctions.^114,115^ Vascular endothelial-cadherin primarily is associated with cell-to-cell adhesion.^116,117^ It is a homo-dimeric transmembrane protein whose extracellular domain can connect with the other same molecules of neighboring endothelial cells in the paracellular cleft, and the cytoplasmic domain can interact with the actin filaments via scaffolding proteins.^2^ In the meantime, catenins, p120, plakoprotein are responsible for supporting physics and regulating junctions by forming a bridge that interacts with zonula occludens-1 (tight junctions proteins) and the actin filaments.^118,119^ Besides, other proteins, such as platelet and endothelial cell adhesion molecule 1, CD99, and nectin, have been reported that might be related to the adherens junction.^120–122^ Totally, adherens junctions are fundamental for the integrity of BBB, any change of adherens junctions may disrupt inter-endothelial cell connections.^123^

### Physiology of the BBB

The existence of BBB provides a controlled microenvironment by regulating the exchange of ions and molecules between the bloodstream and brain tissue. Numerous studies have revealed the physiological functions of the BBB such as brain protection. In addition to physical restriction of the potentially harmful substances, the BBB also plays multiple roles in maintaining homeostasis,^124,125^ facilitating transport of the essential molecules,^126^ regulating inflammation^127,128^ and so forth. The BBB maintains the brain homeostasis by regulating the specific ions channels and transporters. For example, Na^+^, K^+^, Ca^2+^, Cl^−^ are the major ions in the CNS, which should be kept at an optimal level for neural and synaptic signaling functions. These ions are asymmetrically distributed between luminal and abluminal membranes, of which efflux and influx were mainly dependent on the ion transporters on BBB. For example, the influx of Na^+^ and the efflux of K^+^ are regulated by abluminal Na-K-ATPase against the concentration gradient for maintenance of the electrochemical gradient across the cell membrane.^129^ Meanwhile, cotransporters such as NKCC1 regulate the ion balance by transporting Na^+^, K^+^, Cl^−^.^130^ The dysfunction of the ion transporters may induce the pathological alterations. Other BBB transporters such as solute carriers and ATP-binding case families control the transport of other essential molecules, metabolites and nutrients, maintaining the brain homeostasis.^131^ Intriguingly, pathways through the junctional complex or across the cells actively that participate in transport of ions, nutrients and other molecules could also be potential routes for drug delivery, which is discussed in detail in Section 3.1.

## Strategies for BBB regulation and crossing

Brain tumors, cerebrovascular diseases, and neurodegenerative diseases, including Parkinson’s disease, Alzheimer’s disease, and multiple sclerosis, are serious CNS diseases.^132^ However, therapies for these challenging diseases are limited, because of the lack of effective methods, to enable drugs surpass natural protective hindrances to maintain homeostasis within the brain for preventing the entry of drug molecules to the CNS.^133^ A number of drugs are routinely administrated by invasive strategies for the management of these diseases and symptom control. For instance, intrathecal drug administration is a typical method for drug delivery into the entire ventricular system without passing through the BBB.^134,135^ It has been approved to deliver anti-sense oligonucleotides for the treatment of spinal muscular atrophy.^136^ Other invasive strategies including convection-enhanced delivery,^137,138^ intracranial implantation,^139,140^ and deep-brain stimulation were developed for brain drug delivery.^141,142^ Recent development of microneedle and polymeric wafers with minimal invasiveness provides sustained drug release for CNS disease management which circumvent the BBB.^143–146^ But the potential risks from brain exposure and damage of these invasive strategies limited their applications for long-term use. In order to efficiently deliver drugs into the brain with safety and precision, various non-invasive strategies to overcome BBB have been exploited. These strategies include BBB penetration via passive transcytosis, intranasal administration, ligands conjugation, membrane coating, and the BBB disruption using light, focused ultrasound, biochemical reagents, and radiation.

### Passive transcytosis

Passive transcytosis, also known as non-specific transfers, has two possible routes across the microvascular endothelial layer, via paracellular and transcellular.^147^

The paracellular pathway is the principal route blocked by the tight junctions of cell gaps, restricting ions, polar solutes, and most macromolecules.^148^ But the tight junctions are not perfect, small and soluble substances could sufficiently cross through the paracellular pathway.^149^ Typically, BBB is destroyed in some brain diseases. One strategy for therapeutics to use the paracellular pathway is by downregulating the expression of tight junction proteins. For example, a modulator called minoxidil sulfate (MS) that anchored the potassium channel, enhances shipping by attenuating the tight junction proteins.^150^ Zhou et al. developed brain tumor-targeting ligand-modified nanoparticles named CTX-mHph2-III-62%, inside which co-encapsulation of three modulators, minoxidil, lexiscan, and NECA.^151^ If without further engineering, the ability of terpolymer III-62% to penetrate the BBB is limited. Through co-encapsulation, such nanoparticles could release BBB modulators in the local tumor site, which adjust the permeability of BBB in the paracellular pathway to ensure more nanoparticles in the same area. Besides, Han et al. developed M@H-NPs using hyaluronic acid-based nanoparticles to load minoxidil, which can target brain metastatic tumors.^152^ Hyaluronic acid can specially target cell surface receptor CD44, which is highly expressed in breast cancer.^153^ With the help of the CD44 target and MS boost, M@H-NPs could penetrate blood–brain tumor barrier (BTB), internalizing into brain metastatic tumor cells (BMTCs), blunting drug efflux at BMTCs, and producing effective treatment which can extend the median survival time in breast cancer brain metastases models.

The transcellular pathway is the preferable route for carriers to enter and transport therapeutic compounds, when compared to the paracellular pathway. In brief, the molecules can partition into the cellular membranes from the top to the bottom lateral side through carrier-mediated and receptor-mediated transcytosis.^154^ In particular, lipophilic carriers and some other known carrier systems like cationic amino acids are mainly transported via the transcellular pathway. For instance, Allan et al. showed targeting is possible in intracerebral hemorrhage (ICH) using lipid nanoparticles.^155^ They fabricated a liposome HSPC:CHOL: DSPE-PEG2000 and injected them into ICH mice. Brain liposome accumulation was measured via radioisotope and optical detection methods, in order to investigate the kinetics of the liposome across the brain. From their results, after injection, the entry of liposome amounts peaked at 3 h and 48 h, which provided evidence that by utilizing transcellular pathways, liposomes can gather at the lesion site through self-diffusion. Gu et al. modified OX26 antibody to the surface of PEGylated cationic lipid nanoparticles to load baicalin, which was known as OX26-PEG-CSLN.^156^ OX26-PEG-CSLN not only can deliver drugs across BBB but also can adjust the changes of extracellular amino acids. OX26-PEG-CSLN showed a stronger effect than the baicalin solution., which might be attributed to the more enhanced ability to penetrate the BBB and prolong the valid time of the drugs.

The tightness of the BBB precludes the entrance of most pharmaceuticals into the brain via passive transcytosis, except for hydrophilic compounds which have a mass lower than 150 Da and highly hydrophobic compounds which have a mass lower than 400–600 Da.^157,158^ For this reason, if passive transcytosis is demanded to realize the delivery of the drugs at higher molecular weight, some strategies to enhance the BBB permeability or break BBB temporarily will be desired.

### Intranasal administration

**Fig. 2:**
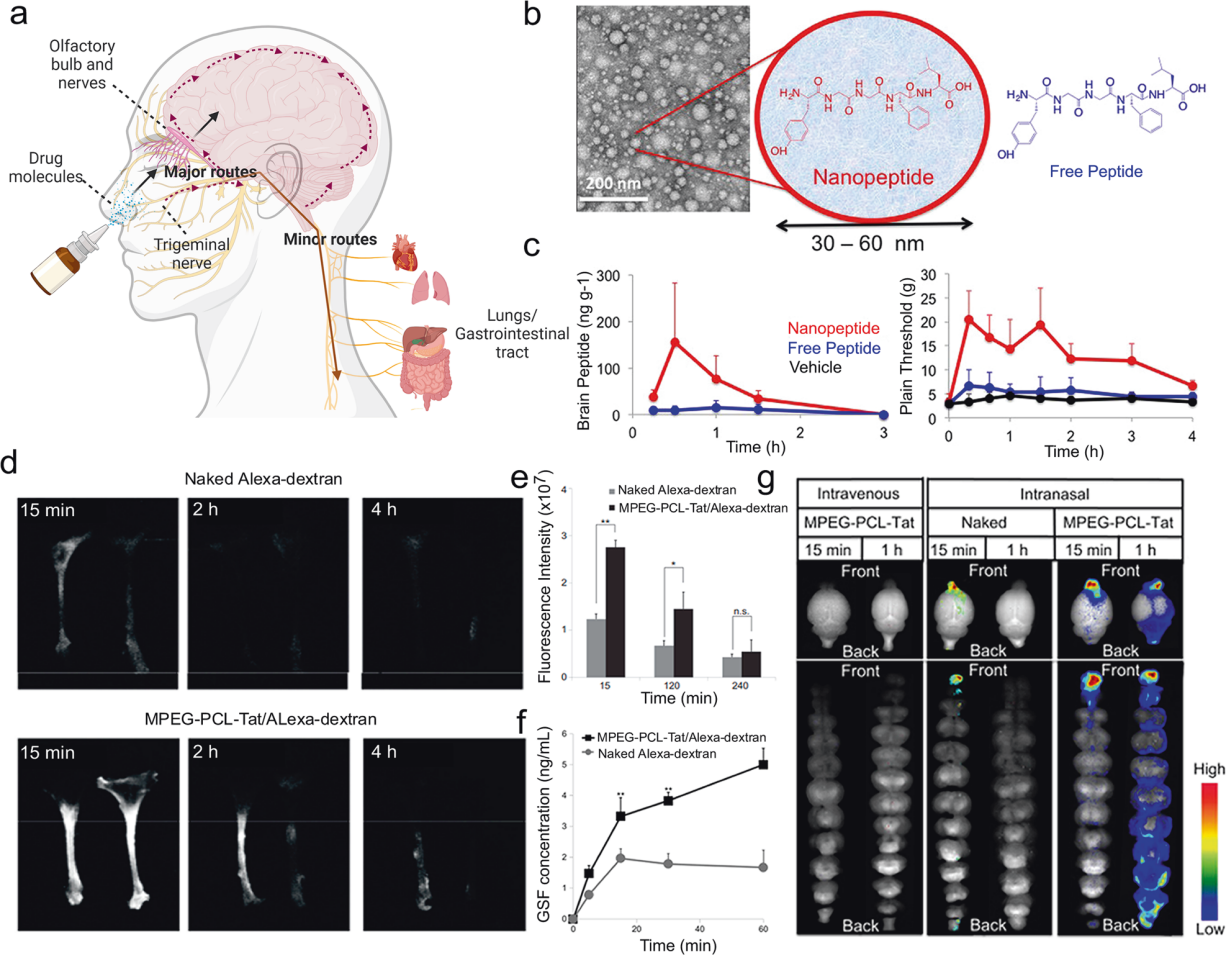
Intranasal administration allows rapid brain targeting from nose to brain. **a** Schematic illustration of the routes from the nasal cavity to the brain. **b** Transmission electron microscopy and structure schematic images of LENK nanoparticles. **c** The change of concentration of LENK in the olfactory bulb and cerebrum after the administration of nanopeptide or as the peptide alone. **b**, **c** Reproduced with permission. Copyright 2017, Elsevier. **d** Dynamics of fluorescence in rat trigeminal nerve. **e** Fluorescence intensity in the trigeminal nerve. **f** CSF concentration of Alexa-dextran. **g** Dynamics of MPEGePCLeTat complex in brain tissue. Reproduced with permission. **d**–**g** Reproduced with permission. Copyright 2013, Elsevier

Intranasal administration is a non-invasive route, allowing rapid brain targeting from nose to brain, when compared to direct injection by intraventricular and intraparenchymal into brain tissue.^159^ Except for those cleared drug molecules by mucociliary clearance, the remains of drugs entered the nasal cavity via neuronal pathway and systemic circulation. As depicted in Fig. 2a, the perineural and perivascular regions of the olfactory and trigeminal nerves are major contributors to the process of the target after intranasal administration. There are three nose-to-brain pathways: (1) Olfactory nerve-olfactory bulb-brain; (2) Trigeminal nerve-brain; and (3) Lungs/ Gastrointestinal tract-blood–brain.^160^ Among them, (1) and (2) are the major routes through neuronal pathways while (3) is the minor route through the systemic circulation. Specifically, (1) is the shortest and most direct route. It has been used advantageously to deliver drugs at high speed. It was possible to complete the delivery within the time window of 1.5-6 h, and even the route via olfactory epithelial cells takes only a few minutes.^161^ (2) is also a direct route of drug delivery, because the trigeminal nerve locates in not only the respiratory region but also the olfactory region. The trigeminal nerve has three branches, each of them joined to the brain stem and olfactory bulb, mainly liable for sensating pain and temperature.^162^ (3) is an indirect route connected to the systemic circulation involving the gastrointestinal and respiratory systems. It belongs to systematic circulation, which may cause extensive drug metabolism. To sum up, the amount of indirect delivery of drugs, when compared to direct delivery, is probably less, due to unexpected elimination in the body.

Intranasal drug delivery systems, as one of the important brain-targeted systems, possess the ability to pass through the BBB using above discussed nose-to-brain pathways. Uchegbu et al. constructed a nano-peptitde with a 30–60 nm particle size, encapsulating leucine5-enkephalin hydrochloride (LENK), and proved this nanoparticle was able to transport LENK through intranasal administration (Fig. 2b, c).^163^ The results presented that after administered, LENK was found in the olfactory bulb, but into the brain it was hard to find them. But using the formulations of nanoparticles, the brain distribution of LENK was facilitated, with no peripheral exposure, and within the thalamus and cortex, nanoparticle localization can be observed. Similarly, in another study, Seta et al. developed nano micelles MPEG-PCL-Tat for intranasal administration using a cell-penetrating peptide (Tat), who is derived from HIV, to modify nano micelles, which were comprised of polyethylene glycol-polycaprolactone polymers (Fig. 2d–g).^164^ Functionally, this system played an important role to deliver siRNA to the brain. The authors harvested nasal olfactory mucosa or olfactory bulb, and prepared frozen samples of that, demonstrating the pathway of nucleic acid transfer using the system, focusing on the major nose-to-brain pathways involving olfactory nerve and trigeminal nerve.

Due to the unique anatomical relationship between the CNS and the nasal cavity, relatively quick along with easy access for nanodrugs to the brain could be realized via intranasal administration. However, there are also some limitations, including the difference in the shape of respective nasal cavities, the exact dosing of intranasal drugs, the mucociliary elimination, and the drainage to the pharynx or to the lower part.^165^ Moreover, the health status of the body also needs to think, otherwise, maybe someone occurs conditions such as allergies or colds, which is not suited for intranasal administration. It appears the outcomes of delivery by the nasal route differ widely between the studies.^166^ Thus, the nasal route to deliver drugs into the brain is rather immature and more high-quality drug delivery systems should note to solve the above-motioned limitations.

### Ligands conjugation for brain targeting

**Fig. 3:**
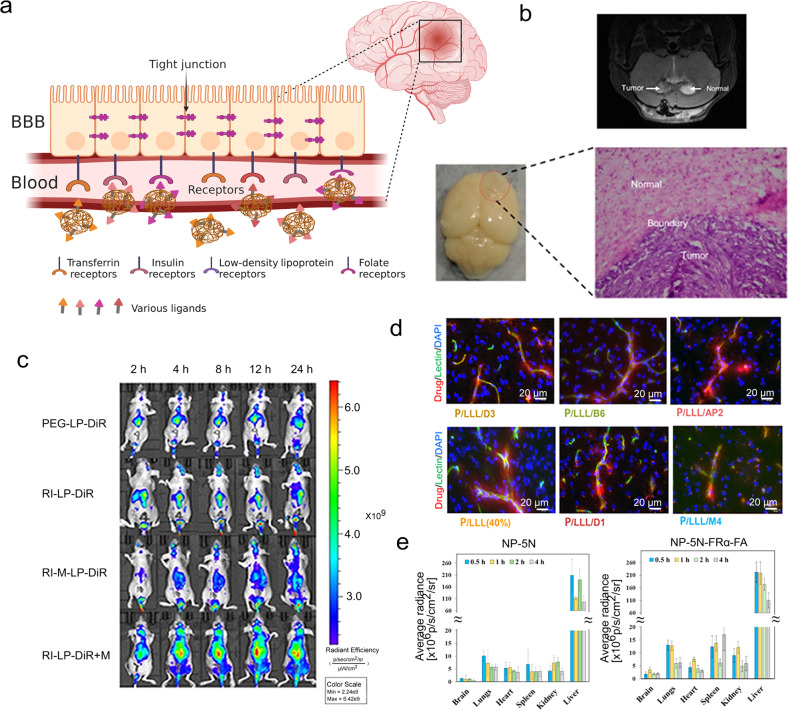
Ligands conjugation is an active targeting strategy using ligands that have high specificity toward the receptor on the endothelial cells of the brain. **a** Schematic illustration of receptor-mediated drug delivery using ligands conjugated nanoparticles to the brain. **b** The MRI images of the brain of glioma-bearing mouse. The photo and micrograph image of glioma which was removed from U87-MG glioma nude mice. **c** Real-time imaging of the U87-MG glioma nude mice. **b**, **c** Reproduced with permission. Copyright 2020, Ivyspring International Publisher. **d** Microscopic visualization of nanoconjugates after permeating the BBB. Scale bar: 20 μm. Reproduced with permission. Copyright 2022, American Chemical Society. **e** Biodistribution results of NIR-797-labeled NP-5N and NP-5N-FRα-FA in ICR mice. Reproduced with permission. Copyright 2019, American Chemical Society

Ligands conjugation is an active targeting strategy using ligands that have high specificity toward the receptor expressed on the brain endothelial cells.^167^ As shown in Fig. 3a, here we take transferrin receptors, insulin receptors, low-density lipoprotein receptors, and folate receptors as examples.

Transferrin receptor as a glycoprotein, has two subunits of 90 kDa linked by a disulfide bridge, participating in the transcytosis of cellular iron by each subunit is capable of binding to one molecule of transferrin.^168^ There have been a lot of studies undertaken using transferrin ligands. Rao et al. formulated a di-block polymer of Poly-lactic-co-glycolic acid (PLGA) and hetero bi-functional COOH-PEG-NH_2_, embedded with an imidazotetrazine alkylating agent (TMZ), and conjugated to a ligand (polysorbate-80/transferrin) and a stem cell targeting moiety (anti-nestin antibody).^169^ Such nanocomposites having targeting ligands could deliver TMZ to intracerebral glioblastoma xenografts and present favorable pharmacokinetics and anti-cancer potential. Qi et al. used RI7217, a monoclonal antibody from mouse, which shows high selectivity and sensitivity for the transferrin receptor, to modify long-circulating liposomes (Fig. 3b, c).^170^ In their research, hCMEC/D3 cells and U87-MG glioma cells were used to evaluate the uptake and mechanism of the targeted liposomes, and intracranial U87-MG glioma was used to test the capacity of the targeted liposomes to cross the BBB and anti-tumor. They concluded that RI7217 antibody decoration is a promising strategy to make a drug delivery system towards brains at the end. Xie et al. optimized dual-mediated liposomes with transferrin and cell-penetrating peptide.^171^ Firstly, in order to construct the rational dual-mediated liposomes, they screened the different PEG molecular weight which is used for connecting transferrin and cell-penetrating peptide (CPP) with liposomes and the densities of ligands. Then, they evaluated the permeability for the BBB of the liposomes to confirm the role of transferrin, and the behaviors of cellular internalization and lysosomal escape to confirm the role of CPP. Meanwhile, nude mice were used as models to trace Tf-CPP-SSL in vivo, demonstrating this drug delivery system performed well in brain targeting and prolonged circulation. However, it should be noted that ligands could not be easily separated from the transferrin receptor and the internalization by endo/lysosomes also compromised the detaching. For this reason, Gao and his group developed a series of nanoplatforms on the basis of acidic cleavable ligand modification.^172,173^ For example, acid-sensitive imine linker (DAK) was conjugated with D-T7 peptide on the nanoparticle surface, which would break up in acidic environment, facilitating the endo/lysosomal escape.^174^ The system was demonstrated to have significant BBB transcytosis enhancement and was employed for further use in the treatment of autism spectrum disorder. Such responsive strategy mitigates the defects of transferrin receptor-mediated transcytosis.

Insulin receptors are highly expressed in the brain, not only in the hypothalamus, olfactory bulb, hippocampus, and striatum, but also in cerebral cortex, and cerebellum.^175^ Insulin could bind to the α-subunits of insulin receptors which locate outside of the cells, inducing the β-subunits which locate in the inside of the cells dimerization and autophosphorylation.^176^ When compared to insulin, insulin-like growth factor 1 (IGF1) showed relative lower binding affinity to insulin receptors.^177^ Similar intracellular signaling pathways could be initiated by both insulin and IGF1 receptors. Jörg et al. employed human serum albumin (HSA) nanoparticles to couple insulin or an anti-insulin receptor monoclonal antibody covalently.^178^ They used loperamide as delivered drugs inside HSA and evaluated the potential of loperamide across the BBB. Loperamide-loaded and insulin-modified HSA presented an increase in size because of the existence of insulin agglomeration on the surface, demonstrating the successful preparation of such insulin-targeting nanoparticles using NHS-PEG-MAL5000 crosslinker. After being injected through the tails of ICR (CD-1) mice, the NP showed significant antinociceptive effects which means loperamide was able to be transported across BBB. Frey II et al. investigated the delivery of IGF1 to CNS, confirming they reached CNS target sites of rats by administering a mixture of [^125^I]-labeled IGF1.^179^ The results from high-resolution phosphor imaging autoradiography established the specific binding of IGF1 and binding sites. Further, they proved IGF1 could activate different signaling pathways in diverse CNS areas.

Low-density lipoprotein receptor-related protein (LRP) as a transmembrane glycoprotein can mediate the uptake of cholesterol-rich low-density lipoprotein, including cholesterol, tocopherol, and Apos.^180^ The family of LRP has a lot of members, such as LRP-1, low-density lipoprotein (LDL) receptor-related protein 1B, megalin/LRP-2, apolipoprotein E receptor 2, sortilin-related receptor, LRP-5, and LRP-6.^33^ Previously, our group has reported an angiopep-2 (a dual-targeting ligand towards LRP-1) modified nanogel for targeted delivery of anti-epileptic drugs.^181,182^ The brain accumulation of electro-responsive nanogels was significantly improved by the functionalization. Interestingly, although the mechanism remains to be explored, the nanogel was found to distribute at temporal lobe which is the common brain region of epileptic focus. Chung et al. synthesized nanoparticles conjugated with angiopep-2 which have core-shell structure, to target and treat glioma.^183^ They proved the decoration of angiopep-2 can improve selective glioma targeting for the amounts of cellular uptake of nanoparticles by C6 glioma cells were higher than that by L929 fibroblasts. Besides, when to contrast the control group, intravenous injection of these angiopep-2 decorated nanoparticles could achieve a 10-fold diminution in tumor volume. Holler and co-wokers reported six peptide vectors were attached to a poly(β-l-malic acid)-trileucine polymer (Fig. 3d).^184^ These peptides could act the specific targeting function, aiming at low-density lipoprotein receptor-related protein-1, transferrin receptor, bee venom-derived ion channel, and Aβ/LRP-1 related transcytosis complex. They studied the ability of nanoconjugates to cross the BBB extensively, including tumor-bearing brains, Alzheimer’s disease-like brains, and healthy brains. What’s more, they conducted molecular mechanisms about regulating cross-talks between transcytosis pathways.

Folate receptors, high-affinity receptors, could mediate the cell uptake of folate or folic acid (FA), anticipating DNA synthesis and nutrient provision.^185^ They are in relatively common use in the design of drug delivery systems. Zhang et al. demonstrated a grapefruit-derived nanovector (GNV) coated with folic acid to carry miR17 for treating the GL-26 brain tumors which is one kind of the folate receptor-positive tumors.^186^ They found GNV reduces the toxicity of the polyethylenimine, which was used for the load of RNA, and FA-GNVs show better targeting behavior than GNVs with rapid movement into the brain within 1.5 h. Shu et al. constructed a folate-modified polymeric micellar delivery system using the thin-film hydration method.^187^ The delivery drug was pterostilbene (Pt) and the main structure of this system was contributed by mPEG-PCL. Compared to free Pt/mPEG-PCL, FA-Pt/mPEG-PCL showed enhanced toxicity toward folate receptors-overexpressing A172 cells, which proves folate anticipated in the condensation of Pt in A172 cells through folate receptor-mediated route. Additionally, in vivo, they analyzed the BBB penetration value and drug targeting index, illustrating the developed delivery system had great potential for brain delivery. Sosnik et al. produced poly(ethylene glycol)-b-poly(ε-caprolactone) block polymers and functioned them in the edge with folate receptor alpha (FRα) and FA (Fig. 3e).^188^ Modified nanoparticles showed better compatibility and greater internalized extent by primary human choroid plexus epithelial cells. After intravenous administration, the biodistribution of unmodified nanoparticles and FRα-FA-modified nanoparticles was tested. As results showed from the systemic circulation, the introduction of FRα and FA to the surface of nanoparticles promote brain accumulation.

### Membrane coating for brain targeting

**Fig. 4:**
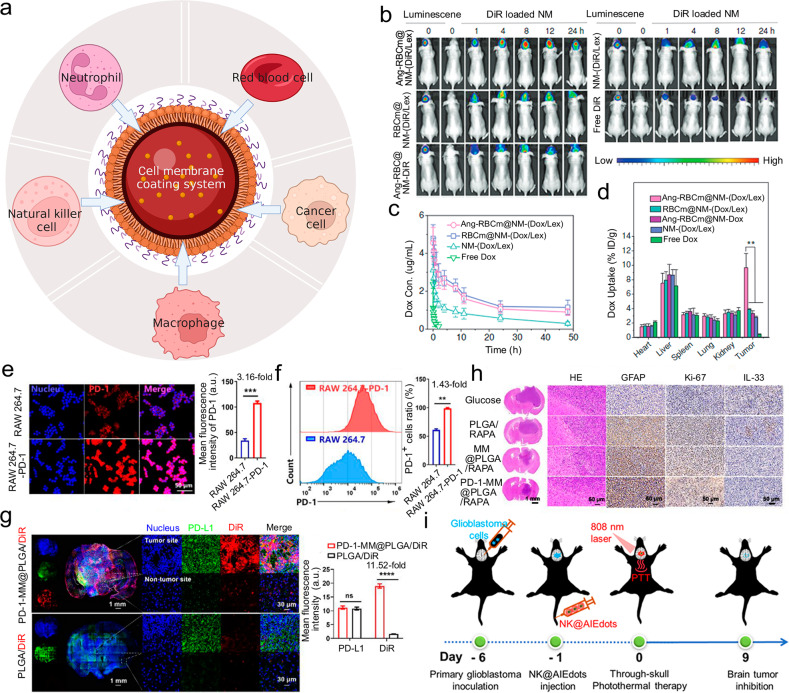
Cell membranes donate drug delivery systems with the brain targeting abilities. **a** Schematic illustration of examples of cell membrane coating strategies. **b** The fluorescence images of orthotopic U87-Luc glioblastoma tumor. **c** In vivo pharmacokinetics. **d** Quantification in different organs and tumor of (doxorubicin) DOX accumulation. **b**–**d** Reproduced with permission. Copyright 2018, Wiley-VCH. **e** The analysis of PD-1 levels on different phenotypes of macrophages. **f** The analysis of the PD-1+ cell ratio in different types of macrophages. **g** The fluorescence images pictured in groups of PLGA/DiR and PD-1-MM@PLGA/DiR. **h** Immunohistochemistry staining images. Scale bar: left 1 mm; right 50 μm. **e**–**h** Reproduced with permission. Copyright 2022, American Chemical Society. **i** Schematic illustration of the process of NK@AIEdots to inhibit the growth of the brain tumor. Reproduced with permission. Copyright 2020, American Chemical Society

Recently, cell membranes as a new root of materials have gained wide focus. They have unique characteristics coming from their parent cells, including the natural functionalities and transmitting information networks that could conquer many obstacles confronted in vivo.^23^ Thanks to the multiple molecular interactions (hydrogen binding, electrostatic interaction, π interaction, etc.) and specific receptor recognition, between the membranes and potential substrates, the membranes of interest could be serve as the outer layer of the delivery systems.^189–191^ For this reason, many drug delivery systems based on different cells’ membrane coating, such as red cell membrane, brain tumor cell membrane, immune cell membrane, and so on, could endow the brain targeting abilities (Fig. 4a).^192–194^

Red blood cells, or erythrocytes, have been exploited as delivery systems due to the advantages of prolonging the life span of drugs in circulation and preventing drugs from immune clearance for many years.^195^ They are easy to obtain and have uniformed size and shape. CD47 proteins rich on the red cell membrane ensure red cell membrane-coated systems can circulate almost 100–120 days without being cleared by macrophages.^196^ Lu et al. used a facile method of avidin-biotin chemistry to modify the red blood cell membrane with CDX peptide, which shows high bond towards nicotinic acetylcholine receptors.^197^ They demonstrated successful preparation of the modified blood cell membrane-coated systems and loading of DOX. And they verified the red cell membrane coating could improve the circulation time of the systems and locate them close to tumor vessels. Both targeting and therapeutic efficiency studies in cells and in animals illustrated that red blood cell membrane-peptide-coated systems not only had the capability to traverse the BBB, displaying exceptional brain targeting effect, but also could release DOX and prolong the survival of mice. Shi’s group developed a biomimetic nanoparticles by modifying angiopep-2 to the surface of red blood cell membranes to camouflage polymer which was pH-sensitive and coload with anti-cancer drug DOX and BBB regulator lexiscan (Fig. 4b–d).^198^ The system with low immunogenicity and systemic toxicity improved blood cycle time and tumor accumulation in U87MG glioblastoma tumor-bearing nude mice.

Brain tumor cell membrane, originated from brain tumor cells, tend to have homotypic targeting, long-time circulation, and BBB crossing abilities.^199^ Specific membrane proteins, such as focal adhesion proteins, integrin, focal adhesion kinase, and ras homologous family proteins, contribute to the functions of brain tumor cell membrane-coated systems.^200^ Liu et al. fabricated lanthanide-doped nanoparticles with the coating of brain tumor cell membrane, which can be used for brain tumor visualization and surgical navigation in the window from 1500 nm to 1700 nm in near-infrared-IIb.^201^ Because of the existence of the cell membrane from the brain tumor, this nanoparticle could easily home to the tumor site. They compared the particle with indocyanine green which is a clinically approved imaging agent, and discovered this particle had a superior resolution with lower background signals, offering a clear view of the location of the tumor.

Immune cell membranes, like their mother immune cells, are a promising choice for constructing drug delivery systems, showing great biocompatibility and unnoticeable adverse effects to normal cells.^202^

Macrophages play important part in the physiological microenvironment and the polarization of their phenotypes affects tumor progression and metastasis directly. In response to neuroinflammation, macrophages could be activated into anti-inflammatory state, serving as protectors digesting cell debris and pathogens, as well as releasing anti-inflammatory factors and activating other immune cells. Thus, the macrophage-based strategy for drug delivery harnesses the prolonged circulation time, abundant surface receptors, and active targeting ability of macrophages under specific phenotype.^203^ Wang et al. enhanced programmed cell death-1 expression on macrophage membranes and coated them onto rapamycin (RAPA)-loaded PLGA core to fabricate a novel nano-platform (PD-1-MM@PLGA/RAPA) (Fig. 4e-h).^204^ Macrophage membranes help the nano-platform to travel across the BBB in response to multiple chemokines. Programmed cell death-1 expression on macrophage membranes optimized the efficacy of immunotherapy, due to the PD-1/PD-L1 signal axis blockade. RAPA, as model drug, could induce cancer cell death and complement immunotherapy. This novel nano-platform provided an anti-glioblastoma multiforme (GBM) strategy through the combination of chemotherapy and immunotherapy. Their results showed more tumor-infiltrating immune-stimulatory cells, especially CD8^+^ cytotoxic T-lymphocyte, were recruited and triggered the release of anti-tumor cytokines, magnifying the anti-tumor effect. Sun et al. attached rabies virus glycoprotein and triphenylphosphine cation molecules on the macrophages membrane and coated them to solid lipid nanoparticles (SLNs), constructing RVG/TPP-MASLNs for delivering natural antioxidants Genistein (GS) to neuronal mitochondria which is a new curative target for Alzheimer’s disease.^205^ The MA membranes provided RVG/TPP-MASLNs with favorable biocompatibility and reticuloendothelial system evasion behaviors. In addition, the combination of MA membranes and functional ligands endowed RVG/TPP-MASLNs with the capabilities for double targeting including neuronal targeting and mitochondria targeting.

Neutrophils are the main type of white blood cells defensing against the pathogens and could migrate from circulation to injured brain region by crossing the BBB. Upon recruitment, the membrane adhesion proteins such as intercellular adhesion molecule-1 on endothelial cells are up-regulated, and the proteins including integrin β2, macrophage-1 antigen, and lymphocyte function-associated antigen 1 are overexpressed on the neutrophil membrane, together facilitating the transmigration.^206^ Chen et al. proposed a “nanobuffer”, (LA-NM-NP/CBD), that has a clear structure.^201^ The inner core consists of PLGA nanoparticles and reactive oxygen species (ROS)-scavenging cannabidiol (CBD). The neutrophil membrane serves as the shell to orient to the infarct core while α-lipoic acid (LA) serves as the corona to scavenge ROS. They used LA-NM-NP/CBD to change the adverse environment of the brain and take care of the salvageable penumbra for the therapy of ischemic stroke.

Natural killer (NK) cells are large granular lymphocytes and can naturally undergo immunosurveillance of diseased/stressed cells.^207^ With the assistance of inhibitory and activating receptors on the cell surface such as LAF-1 and VLA-4, they could efficient target specific cells, providing the potential of brain-targeted delivery. They can directly bind to cancer cells via receptors and kill them without prior sensitization. Tang and co-workers developed nanorobots by coating an aggregation-induced emission-active polymeric endoskeleton with a membrane from NK cells to mimic NK cells (Fig. 4i).^208^ The mechanistic studies demonstrated that receptors from NK cells to the surface of the nanorobots, play a major duty in BBB traversing and tumor identification. Besides, with help of a laser, the AIE-active conjugated polymer could be a tight junction modulator, helping to disrupt the tight junction and making this nanorobot cross the BBB easier.

### External stimuli-mediated BBB disruption

To regulate the BBB permeability, external stimuli-mediated BBB disruption based on the energy conversion materials has been widely explored. Such strategies can be manipulated with various external stimulations, such as light, ultrasound, electroacupuncture, etc.

Light has been widely used due to its advantages, such as spatiotemporal precision, minimized scattering and domestic nonlinear absorption.^27^ As mentioned in the above chapter, light could help to open the BBB, inducing specific changes in the integrity of BBB. This phenomenon is temporary and could recover. In 1990, Eggert et al. investigated the Nd:YAG laser irradiation.^209^ They found laser irradiation immediately caused BBB breakdown which looked to be associated with structural damaged regions of brain microvessels. Besides, it suggested that laser-induced BBB abnormality or impairment is monophasic. After irradiation, the BBB dysfunction peaked at 2 h and persisted for 24 h approximately. However, the effects on brain from the irradiation is highly dependent on power intensity, irradiation time and distance between laser source and targeted area, which in all contributed to the temperature elevation. Thanks to the development of temperature measurement technology, researchers are able to monitor the temperature in a non-contact manner. Among all, near-infrared light (NIR) at the wavelength from 700 to 1600 nm has attracted great attention in this field due to its deep tissue penetration.^210–212^ In recent studies, NIR irradiation for BBB permeability regulation was applied under infrared thermal monitoring and the head temperature was kept lower than 43 °C. At this power intensity, the irradiation caused negligible brain damage confirmed by histological staining.^213,214^ The reduced transendothelial electrical resistance of cellular monolayer induced by irradiation could recover within 10 min.^215^ In 2011, Choi et al. reported without compromising vascular integrity, the ultrashort pulsed laser could induce transient leakage of blood plasma.^216^ They combined the ultrashort pulsed laser with a systemic injection to deliver target molecules to brain cortex and different other tissues. This strategy allowed invasive local delivery to an extremely small extent. In 2018, Guo and co-workers demonstrated that 2D black phosphorus nanosheets that possess excellent photothermal effects can be a new neuroprotective platform to selectively capture Cu^2+^ for treating neurodegenerative disorders.^214^ Under near-infrared irradiation, local hyperthermia for five minutes with the temperature at 41–43 °C increased the generation of tiny mechanical waves thus increasing the BBB permeability. Their results showed that the power density of light could be kept over 37% of the original value and the depth could be ~1.3–2.6 mm for the mouse brain. In addition, they studied the risk of cerebral thrombosis using nuclear magnetic resonance imaging and found no obvious cerebral thrombosis, which means the black phosphorus nanosheets have great potential in future clinical applications. In 2019, Wang and co-workers also designed black phosphorus nanosheets-based drug delivery system for loading with the antidepressant drug, Fluoxetine.^217^ They conducted the release ability of fluoxetine and proved 90% of the drugs could be released under 30 min light irradiation. For in vivo studies, they reported, with near-infrared irradiation, the local temperature was kept at 41–43 °C for five minutes. Finally, they compared free fluoxetine and black phosphorus nanosheets loaded fluoxetine and got the conclusion that black phosphorus nanosheets loaded fluoxetine shorted the therapy time of depression with the help of light. In 2021, Qin et al. modulated BBB by using light boost of molecular targeted nanoparticles, the synthesized gold nanoparticles, which were conjugated with the antibody BV11.^218^ Their results showed after light stimulation of BV11 modified gold nanoparticles, tight junctions of BBB ameliorated, allowing particles like macromolecules and virus to cross. Brain microvasculature and parenchyma were also examined. There were no obvious disruptions in vast dynamics or neuronal injury. Recently, an electro-responsive dopamine-pyrrole hybrid system that improved the delivery efficiency of anti-epileptic drugs by improving the cross of BBB via the combination of receptor-mediated transcytosis and photothermal conversion of NIR were reported.^215^ This system was smart for epilepsy pharmacotherapy, showing enhanced conductivity and sensitivity in various seizure models, including acute seizure, continuous seizure, and spontaneous seizure. The authors realized two hours sustained and 30 s rapid release of phenytoin and reduced drug dosage.

Ultrasound is a technique that can noninvasively focus deep into the body using an ultrasound field.^219^ They are mechanical or elastic vibrations in a medium of a frequency above the range of human hearing (18–20 kHz).^220^ Since the 1940s, ultrasound has been noted for non-invasive ablation in the brain.^221^ For ultrasonic BBB disruption, varied sonication parameters can cause different impacts, including the threshold pressure, the magnitude, and the drug quantity for delivering.^222^ In recent years, gas-filled microbubble has emerged as a contrast agent in conjunction with ultrasound for opening the BBB in an image-guided and targeted manner, ensuring the local delivery of drugs.^223^ Qin et al. constructed a microbubble delivery system, fixing quercetin-modified sulfur nanoparticles.^224^ In combination with ultrasound, this system could accumulate in the brain and promote drug delivery because of the transient opening of the BBB. Moreover, Qc@SNPs-MB effectively treated Alzheimer’s disease by protecting nerve cells and reducing endoplasmic reticulum stress which comes from oxidative stress, inflammatory response, calcium homeostasis imbalance, and neuronal apoptosis. Price’s group utilized gas-filled microbubbles for the selective transfection of endothelial cells of the cerebral vasculature.^225^ The negative mCherry plasmid was conjugated to cationic microbubbles. Such microbubbles have good stability. Their method through the oscillating of microbubbles under the ultrasound field could implement the transport of the gene product beyond the blood vessels without breaking the tight junctions and disrupting the BBB. To be noted, one highlight of this study is the experiments on cell identification and enrichment of whole-brain tissue samples, demonstrating their strategy achieved around 90% cell specificity of selective transfection with no extra use of a cell-specific promoter.

Electroacupuncture is a widely accepted complementary therapy through stimulating acupoints, although there is limited supporting information in modern anatomical studies.^226^ Studies found that electroacupuncture stimulation at certain parameters could improve the permeability of the BBB.^227^ Lin and co-workers found the Baihui and Shuigou acupoints are ideal regions to use for electroacupuncture for 40 min to open BBB.^228^ However, research on the application of nanomaterials in synergy with electroacupuncture in the brain has not yet been found. We believe there will be improvements in this cross-field in the future.

### Other non-invasive strategies

**Fig. 5:**
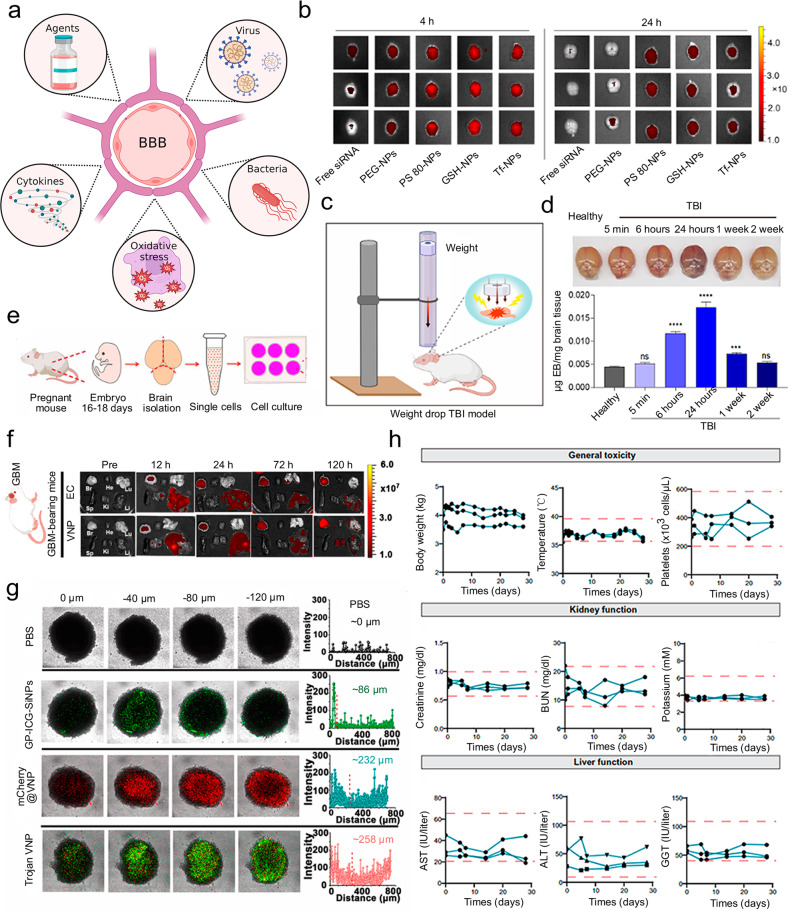
Other strategies can achieve the intention of brain-targeted drug delivery. **a** Schematic illustration of examples of other strategies. **b** Fluorescence images using in vivo imaging system of isolated brains. **c** Schematic illustration of the weight drop-induced TBI model. **d** Time points of physically invaded BBB was studied. **e** Schematic illustration of the isolation of primary neuronal cells from mouse embryos. **b**–**e** Reproduced with permission. Copyright 2020, American Association for the Advancement of Science. **f** Ex vivo fluorescence images of primary organs of glioma-bearing mice. **g** Confocal images and corresponding fluorescence intensity of 3D tumor microspheres. **f**, **g** Reproduced with permission. Copyright 2022, Nature Publishing Group. **h** Evaluation of general toxicity, kidney function and liver function over time. Reproduced with permission. Copyright 2022, American Association for the Advancement of Science

Besides the widely used strategies that have been discussed before, among which active strategies for BBB regulation and crossing are summarized in Table 1, there are more strategies that people used to achieve the intention of brain-targeted drug delivery (Fig. 5a).

**Table 1:** Active strategies for BBB regulation and crossing

Strategy	Material/technique	Advantage	Specificity	Refs.
Ligands conjugation	Transferrin	High specificity	Affinity to transferrin receptor	^169,171^
	RI7217	High selectivity and sensitivity	Affinity to transferrin receptor	^170^
	Acidic cleavable ligand-modified peptide	Facilitating the endo/lysosomal escape	Affinity to transferrin receptor	^172–174^
	Insulin or anti-insulin receptor monoclonal antibody	Highly expressed	Affinity to insulin receptor	^178^
	IGF1	Activate different signaling pathways in diverse CNS areas	Affinity to IGF1 receptor	^179^
	Angiopep-2	High BBB transcytosis capacity	Affinity to LDL receptor	^181–183^
	Six peptide vector	Muti-specific targeting function	Affinity to low-density LRP-1, transferrin receptor, bee venom-derived ion channel, and Aβ/LRP-1 related transcytosis complex	^184^
	Folate	Compatibility and internalized extent	Affinity to folate receptor	^186–188^
Membrane coating	Red cell membrane	Long circulation and preventing from immune clearance	Affinity to CD47 proteins receptor	^195–198^
	Brain tumor cell membrane	Homotypic targeting and long-time circulation	Affinity to focal adhesion proteins, integrin, focal adhesion kinase, and RHO family protein	^199–201^
	Macrophage membrane	Favorable biocompatibility and RES evasion behavior	Polarization of their phenotypes affects brain tumor progression and metastasis	^204,205^
	Neutrophil membrane	Pass across tissues and walls of veins	Affinity to infarct core and inflammatory cytokine	^201^
	Natural killer cell membrane	Directly bind to cancer cells via receptors and kill them without prior sensitization	Naturally undergo immunosurveillance of diseased/stressed cells	^207,208^
External stimulation	Light (NIR)	Minimized scattering, domestic nonlinear absorption, and deep tissue penetration.	Help to open the BBB, inducing specific changes in the integrity of BBB	^209,213–218^
	Gas-filled microbubble/Focused ultrasound	Deep tissue penetration.	Non-invasive ablation	^219–225^
	Electroacupuncture	Improve the BBB permeability	Stimulating acupoints	^226–228^
	Biochemical reagents	Adjust the penetration	Shrink the endothelial cells of the brain	^229,230^
Other active strategies	AAVs	High efficiency	Specific molecules identified on the luminal surface of the BBB	^231,233–236^
	Bacteria	Act as antigens	Target and penetrate	^237,238^
	Redox-sensitive systems and cytokine-mediated system	Responsive and sensitive	Developed for oxidative stress and the pro-inflammatory state	^239–244^

Agents, such as metals, polysorbate-80 (PS-80), etc., are used to shrink the endothelial cells of the brain.^229^ Using this treatment, various drug delivery systems could bypass BBB. However, it has serious disadvantages, for example, compromising the integrity of BBB and further leading unwanted exogenous agents, including blood components, neurotoxic, and xenobiotics, to accumulate in cerebral tissue. For short, it is a non-patient-friendly method that may cause injury to the CNS. Joshi et al. reported a PLGA-based with different surface coatings platforms used for brain delivery of siRNA to treat traumatic brain injury (TBI; Fig. 5b–e).^230^ They tried a nonionic surfactant PS-80 coating among their formulations and proved drug delivery across BBB could be promoted by interacting with lipoprotein receptors. They euthanized mice after injection and extracted brains for observing the fluorescence signals labeled nanoparticles. PS-80 nanoparticles showed significantly superior fluorescence signals than other formulations. They pointed out in their manuscript that although the joint modulation of surface chemistry and PS-80 density, is a useful tool to adjust BBB penetration, functional or behavior evaluations, for instance, cell death, neuroinflammation, or larger animal models, are lacking in the study. Hence, biosafety and performance in clinical may still have questions.

AAVs, presenting targeting ligands, could interact with specific molecules identified on the luminal surface of the BBB.^231^ The integrity of BBB could be breached by the intrinsic properties of the pathogens.^232^ Directed evolution and capsid engineering have been developed to engineer a number of BBB-crossing AAVs.^233^ Among the AAV family, a natural AAV9 capsid variant which was isolated from human liver tissue, has the ability to bypass BBB, becoming a star capsid for drug delivery into CNS.^234^ After intravenous injection, AAV9 mainly enriches the brain and liver.^235^ Bei et al. reported a rational design of two AAV9 variants for brain drug delivery.^236^ Confirmed by rodent and primate models, the variants prepared by insertion of cell-penetrating peptide enhanced both BBB transcytosis and cellular transduction. The authors claimed that these variants not only displayed increased BBB crossing, but also of greater importance maintained the neurotropism, paving the translational potential of neurological disorders treatment.

Bacteria can go through the BBB and infect phagocytes.^237^ Researchers have proved the feasibility of adopting bacteria to build drug delivery systems to resist central nervous system diseases. For glioblastoma photothermal immunotherapy, Sun et al. developed a ‘Trojan bacteria’ consisting of two types of bacteria (Fig. 5f, g).^238^ They demonstrated that intravenously injected Trojan bacteria system could target and penetrate glioblastoma. The confocal images showed that 3D tumor microspheres could be penetrated with a depth of around 260 μm. With the help of a laser, bacterial cells and the adjacent tumor cells could be destructed by the heat from irradiation. The debris from bacteria and tumor cells also can act as antigens to promote cancer immunotherapy.

BBB is also modulated by redox-sensitive systems and cytokine-mediated systems. The former is mainly developed for oxidative stress, which is the similarity between brain disease.^239^ The latter is mainly developed for the pro-inflammatory state, which is a common state in a number of CNS pathologies.^240^ Oxidative stress is incited by an imbalance of oxidants.^241^ These oxidants can further impact a variety of signaling pathways associated with pathological processes, resulting in BBB dysfunction eventually.^242^ Redox-sensitive systems could be designed in response to the high level of oxidants, for example, Kong et al. reviewed the related developments in nervine.^243^ They discussed the choice of ROS-responsive functional groups, including sulfides, selenide, ferrocene, amino acrylate, etc. Meanwhile, the pro-inflammatory state, a result of local oxidative stress, stimulates many cytokines, such as IL-1 and TNF-α. Thus, conducting cytokine-mediated systems is an optional method. Veiseh et al. reported a cytokine delivery platform as interleukin-2-producing cytokine factory organized with polymer and ARPE-19 cells which can be clinically translatable (Fig. 5h).^244^ They conducted this robust platform in peritoneal tumors in ovarian and colorectal mouse models and considered it could be addressed in other cancers including brain cancer. They found no significant deviations from healthy ranges using complete blood count and blood chemistry analysis, showing this system was well tolerated in nonhuman primates.

## Engineered brain-targeted drug delivery systems

To fight against the BBB, a growing number of advanced materials and technologies have been developed for enhanced brain-targeted drug delivery in CNS disorder treatment. By using the passive and/or active strategies for BBB regulation and crossing in a controlled and non-invasive manner, various drug delivery systems are engineered to facilitate the brain-targeted delivery of specific therapeutics including small-molecule drugs, proteins, genes, and other biopharmaceutics. Due to the requirement of both delivery efficiency and safety, the majority of drug delivery systems with the ability to cross BBB are designed at a nanoscale level with tailored chemical composition and surface properties (Table 2). Since the approval of DOX in liposomes (Doxil) for anti-cancer treatment nearly three decades ago, increasing numbers of drug delivery systems are studied in clinical trials and some formulations for enhanced drug delivery have entered to clinic.^245–247^

**Table 2:** Brain-targeted drug delivery systems engineered by various material types

Material type	Size (nm)	Representative delivery cargo	Refs.
Liposome	50–500	cisplatin, curcumin, DOX, erlotinib, obidoxime, paclitaxel, plasmid β-galactosidase	^254,255,257,258,260,383,384^
Micelle	2–300	curcumin, temozolomide, resveratrol, siRNA, RAPA	^268–270,385,386^
Polymeric nanoparticle	<200	enzyme, Epothilone B, memantine, Schisantherin A	^264,387–389^
Nanogel	50–300	DOX, harmine, insulin, thymidine analog, teriflunomide	^272,273,275–277^
Gold nanoparticle	2–80	cisplatin, DOX, insulin, lacosamide, quercetin, siRNA	^290,295,296,298,390–392^
Carbon nanotube	20–200	DOX	^393,394^
Graphene (oxide)	20–1000; thickness: <5	plasmid DNA, puerarin, ruthenium carbonyl clusters	^313,395^
Graphene quantum dot	<20	docetaxel	^318^
Iron oxide nanoparticle	10–300	cisplatin, paclitaxel, siRNA	^332,333^
Mesoporous silica nanoparticle	20–300; pore size: 2–50	berberine, docetaxel, DOX, leptin, monophosphate, pioglitazone, paclitaxel, siRNA, trastuzumab	^341,343,345–347,396^
Biomimetic system	40–200	a-CTLA-4, a-PD-1, chlorin e6, coumarin 6, CPPO, curcumin, dopamine, DOX, glucose polymer, human Mucin 1 protein, indocyanine green, methotrexate, monoclonal antibodies, mRNA, phosphatase, proteins, siRNA, succinobucol	^238,359,360,365–367,369,397–406^
Black phosphorus	3–250; thickness: <20	DOX, matrine, paeoniflorin	^371,407,408^

### Liposomal formulations

Liposomes are the first generation of drug delivery systems and have been widely used since their discovery in 1965.^248^ They are composed of one or more lipid bilayers and hollow aqueous compartment, which endow them with loading versatility for both hydrophobic and hydrophilic therapeutic agents.^249^ Together with high biocompatibility, biodegradability and its intrinsic capability of BBB crossing, liposomes are considered as one of the most successful delivery systems with a great potential in translational medicine. In the past decade, research on liposomes has further substantially increased along with the advance of materials engineering and nanotechnology.^250^ Various types of functionalization strategies have been involved for the development of liposomal delivery system such as brain/tumor-targeting delivery, controlled drug release, imaging-guided delivery, etc.^251^ These strategies facilitate the development of liposomal formulations to improve brain-specific delivery.

**Fig. 6:**
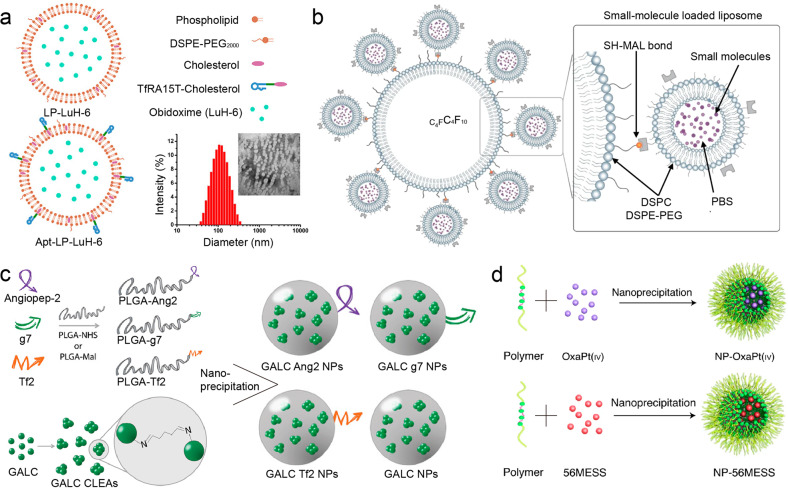
Liposomal formulations and polymeric materials for brain-targeted drug delivery. **a** Schematic illustration of synthesis of Apt-LP-LuH-6 liposomes and the structure characterization. Reproduced with permission. Copyright 2021, Elsevier. **b** Small-molecule-loaded ultrasound-controlled liposomal nanocarrier. Reproduced with permission. Copyright 2020, Nature Publishing Group. **c** Schematic illustration of synthesis of micelles by nanoprecipitation. Reproduced with permission. Copyright 2019, American Association for the Advancement of Science. **d** Schematic illustration of nanoprecipitation of peptide-modified PLGA and GALC CLEAs for the generation of enzyme delivery system. Reproduced with permission. Copyright 2021, Nature Publishing Group

Brain-targeted delivery of the liposomes could be enhanced by modification of targeting ligands including polymers, peptides, antibodies, and aptamers.^252^ For example, Zhan et al. functionalized liposomal surface with amyloid-β-derived peptide which has a highly specific binding affinity towards plasm apolipoproteins, giving a protein corona-modified liposomal system. Significant enhancement of the brain distribution of DOX-loaded liposomes as well as higher anti-tumor efficacy were found.^253^ Quan et al. developed transferrin receptor aptamer-functionalized liposomes to deliver acetylcholinesterase reactivator in brain (Fig. 6a).^254^ Compared with non-targeting liposomes, this functionalized system has higher BBB penetration efficiency confirmed by both in vitro BBB model and in vivo biodistribution study. Conjugation of brain-targeting peptide is also proved to be an effective method to improve the brain accumulation. Zhang et al. used RVG29, a 29 amino-acid peptide derived from rabies virus glycoprotein, as a targeting ligand in the liposome-based delivery system for treatment of Parkinson’s disease.^254^ But it begs for the question that how can targeting ligand-modified liposomes bypass through the BBB via receptor-mediated transcytosis. To give a vivid description, Lauritzen et al. characterized all steps of the liposome delivery routes into brain by using transferrin receptor-targeted liposomal nanoparticles as a model system.^255^ They revealed that post-capillary venules is the key site for transcytosis-mediated brain entry of nanoparticles.

For brain tumor treatment in particular, the systems are required to have a capability of both BBB crossing and tumor targeting.^256^ Thus, dual targeting strategy in liposome functionalization becomes a possible solution for drug delivery in brain tumor therapy. For instance, a GBM-specific cell-penetrating peptide and an anti-GBM antibody were simultaneously anchored onto the liposome surface, giving the ability of BBB penetration.^257^ By incorporation of superparamagnetic iron oxide nanoparticles (SPIONs) and DOX, the liposomes displayed a thermo-responsive drug release in an alternating magnetic field. Another example of dual functionalized liposomes was achieved by Singh’s group.^258^ Surface modification of transferrin and a cell-penetrating peptide was performed. Their liposome system thus showed around 12 and 3.3-fold increases of two chemotherapeutics (DOX and erlotinib) delivery, respectively compared to the free drugs. These strategies confirmed that ligand modification for BBB and tumor targeting could largely improve the drug delivery for brain tumor treatment. Interestingly, the liposome without any targeting ligands conjugation could also serve as an external stimuli-controlled nanosystem for brain-targeted drug delivery. As shown in Fig. 6b, Yanik et al. prepared drug-loaded liposomes tethered to lipid microbubbles containing perfluorobutane gas core.^259^ By applying two-component aggregation and uncaging focused ultrasound sequences at different stages, the drug-loaded liposomes could aggregate locally first and then uncage the cargo responsively to achieve high target specificity. The released drugs can cross the intact BBB without compromising the integrity.

Recently, some new types of liposomes with unique structures were reported for enhanced therapy of CNS diseases. Gomes et al. proposed a concept of exosome-like liposomes to overcome the limitations of large size and low productivity. A lipid film including DODAP (1,2-dioleoyl-3-dimethylammonium-propane) and DPPC (dipalmitoylphosphatidylcholine) was introduced in the preparation of the liposomes, giving the bilayer similar to the outer membrane of exosome.^260^ More liposome-based biomimetic nanomaterials for drug delivery will be emphasized in section 4.7.

### Polymeric drug delivery systems

Polymeric materials for brain-targeted delivery refer an extensive number of drug delivery systems.^261^ They have shown potentials in pre-clinical study in different animal models of CNS diseases demonstrating attractive properties for drug delivery including controlled drug release, cellular targeting and uptake, and the ability to avoid phagocytosis of reticuloendothelial system. PLGA is a typical Food and Drug Administration (FDA)-approved polymer used for the formulation of delivery systems in biomedicine.^262^ PLGA-based delivery system is qualified to encapsulate a wide range of therapeutic cargos including small-molecule drugs, genes, proteins, vaccines, ensuring high bioavailability by protecting them from degradation. The PLGA assembles without any functionalization have a high distribution up to 16.4% in the brain and can keep at the level for at least seven days.^263^ Moreover, the possibility of surface decoration of targeting ligands could further promote their penetration of biological barriers. A typical example was reported by Cecchini et al., demonstrating that PLGA polymers conjugated with different targeting peptides could be used for enzyme therapy of brain disorders such as Krabbe disease (Fig. 6c).^264^

Amphiphilic polymers are known for their advantage of self-assembly into micelles which has a hydrophobic core and a hydrophilic shell at a size of 2–300 nm. Such architecture thus enables high loading capacity and prolonged blood circulation of therapeutic agents, and its efficient accumulation in brain lesions. We recently proposed a micelle-based drug delivery nanosystem for febrile seizure control.^265^ The designed copolymer of poly(acrylamide coacrylonitrile)-methoxy polyethylene glycolsuccinimidyl carbonate could self-assemble into defined micelles. Because of the incomplete development of BBB in mice pups, the micelles could quickly accumulate in neonatal brain as early as five minutes. Anti-inflammatory small-molecular inhibitor CZL-80 was chosen as a model drug. After micelle encapsulation, therapeutic window of the drugs was significantly increased to at least 4 hours, whereas 20 min for injection of CZL-80 alone. Importantly, the nano-engineered micelle is gifted for an upper critical solution temperature of 39 °C, showing a thermos-sensitive drug release mechanism for on-demand therapy. To combat against drug-resistant gliomas, Saltzman et al. prepared a reduction-responsive polymeric nanoparticle for co-delivery of oxaliplatin (the third-generation platinum anti-cancer drug) and 56MESS (a cationic platinum DNA intercalator) (Fig. 6d).^266^ Oxaliplatin and 56MESS were encapsulated inside the nanoparticles through hydrophobic interaction and electrostatic complexation, respectively. Higher level of glutathione (GSH) in tumor reductive microenvironment could trigger the drug release due to the rupture of disulfide bonds in polymer backbones. The authors claimed that by using convection-enhanced delivery, the drugs could traverse the BBB and accumulate at the glioma region. Another case of micelle-based drug delivery system for CNS disease treatment without any brain-targeted functionalization was reported by Jiang’s group.^267^ They developed an microthrombus-targeted micelle-based system for ischemia stroke therapy. Due to the pathologically damaged BBB, microthrombus-targeted micelle could penetrate BBB efficiently and promoted RAPA delivery via hydrophobic interactions. These micelles are assembled by ROS-responsive and fibrin-binding polymer, giving extended drug retention and ROS-triggered drug release for neuroinflammation regulation. Micelle-based delivery systems is also effective in the treatment of those diseases with intact BBB structure. A brain and microglia dual targeting nanosystem was constructed by an targeting peptide derived from β-amyloid protein and ROS-responsive amphiphilic polymer.^268^ By mimicking the unregulated Aβ transportation, the micelles could target the Alzheimer’s disease microenvironment and release the model drug curcumin in response to excessive ROS generation in Alzheimer’s disease. Similar micelle-based strategies with surface modification of targeting ligands were performed for the synergistic chemotherapy of glioma,^269^ gene therapy of Alzheimer’s disease,^270^ and traumatic brain injury.^230^

Nanogel is formulated by three-dimensional crosslink of functional polymers and has a network capable of efficient drug encapsulation.^271^ Tunable properties such as deformability, nanoscale size and high hydrophilicity make nanogel a candidate for nose-to-brain delivery. Di Carlo et al. manufactured a poly(N-vinyl pyrrolidone)-based nanogel by e-beam irradiation and evidenced this nanogel-based system could provide nose-to-brain delivery of insulin.^272^ Compared with free insulin administration, the drug levels in the anterior and cerebellar regions were significantly increased within 60 minutes. The authors indicated this improvement could be resulted from intranasal delivery via olfactory and trigeminal nerve pathways. Another polymeric nanogel delivery system of teriflunomide-loaded lipid-based carbopol-gellan gum in situ gel was reported by Kokare and co-wokers.^273^ By balancing the interactions of gelling agents, mucoadhesive agents, the nanogel was formulated with lipophilic medicine and achieved two-fold enhancement of drug permeability. The same group further developed paliperidone palmitate poloxamer-guar gum nanogel for schizophrenia treatment by nasal delivery directly to the brain bypassing the BBB.^274^ Wang et al. fabricated calcium ions-triggered harmine in situ nanogel through coupling homogenization and spray-drying technology.^275^ The bioavailability of harmine administrated by nanogel was found to be 25-fold higher than that by oral administration. At the meantime, incorporation of brain targeting motifs allows nanogel transcytosis across the BBB for brain-targeted drug delivery. Morgenroth reported nanogel-based carrier for the intracellular delivery of radiophamaceuticals to brain tumor cells by functionalization of ligands of diphtheria toxin receptor overexpressed in abnormal cerebral blood vessels.^276^ The crosslinked network also contained matrix metalloproteinase substrate, permitting protease responsive drug release. Cell membrane-mimicked nanogel is another typical nanocarrier for BBB crossing. For example, Yu and his group utilized phosphorylcholine nanogels for enhanced GBM chemotherapy.^277^ The phosphorylcholine polymer exhibited long-lasting circulation and achieved higher accumulation in brain tumor tissue, serving as an effective tool for BBB crossing.

There are other polymers-enabled delivery platforms that have been proved for successful brain-targeted delivery including semiconducting polymers, polysaccharides, gelatin and other synthetic or natural polymers.^278–282^ Further investigations on bioavailability, degradability, and bio-interactions of polymers in vivo will facilitate the development in this field.

### Gold nanomaterials

Gold nanomaterials have a number of distinctive advantages over drug delivery for brain diseases.^283,284^ Inert surface chemistry of gold allows limited interactions at nano-bio interface and consequently leads to high cellular and tissue compatibility. They could enter the brain by crossing the brain endothelium in a size-dependent manner. In addition, it is comparatively simple to synthesize the gold nanomaterials with tailored structure and defined size. Although only smaller size (<10 nm) gold nanoparticles after intravenous injection was observed to be distributed in the brain slice.^285^ Sub-100-nm gold nanoparticles can extravasate through the damaged BBB under some pathological circumstance such as brain tumor, cerebral stroke, brain injury and epilepsy. Moreover, gold nanomaterials could be traced by using computed tomography imaging for imaging-guided therapy, evaluating their biodistribution and accumulation in the brain.^286,287^ A variety of applications based on gold nanoparticles have developed for brain disorders treatment thanks to their excellent properties. For example, some gold nanoparticles that have higher absorbance at near infrared biowindow are potential nanogents for photothermal therapy of brain tumor.^288^ In this section, we will focus on the discussion of the design and synthesis of gold nanomaterials-based delivery systems.

Functional ligands or drugs can be stably anchored onto the gold surface via gold-thiol bonding for enhanced drug delivery.^289^ Popovtzer developed insulin-coated gold nanoparticles as endogenous BBB transport system for delivering therapeutics into the brain regions, rich in insulin receptors.^290^ Prior to the modification, thiol-mPEG-COOH copolymer was attached onto the gold nanoparticles via gold-thiol interaction, leaving carboxylic groups for further EDC/NHS conjugation with insulin. They also proved the 20 nm of gold nanoparticles showed highest accumulation in the brain compared with 50 nm and 70 nm nanoparticles. Another example of maze tetrapeptide-anchored gold nanoparticles was confirmed by Guo’s group.^291^ The thiolated biomolecules were anchored onto the nanoparticle surface, achieving brain-targeted delivery and neuroprotection to prevent Alzheimer’s disease. As mentioned, the gold-thiol interaction between gold nanoparticles and functional ligand enabled a simple and effective strategy for nanoparticles functionalization.

**Fig. 7:**
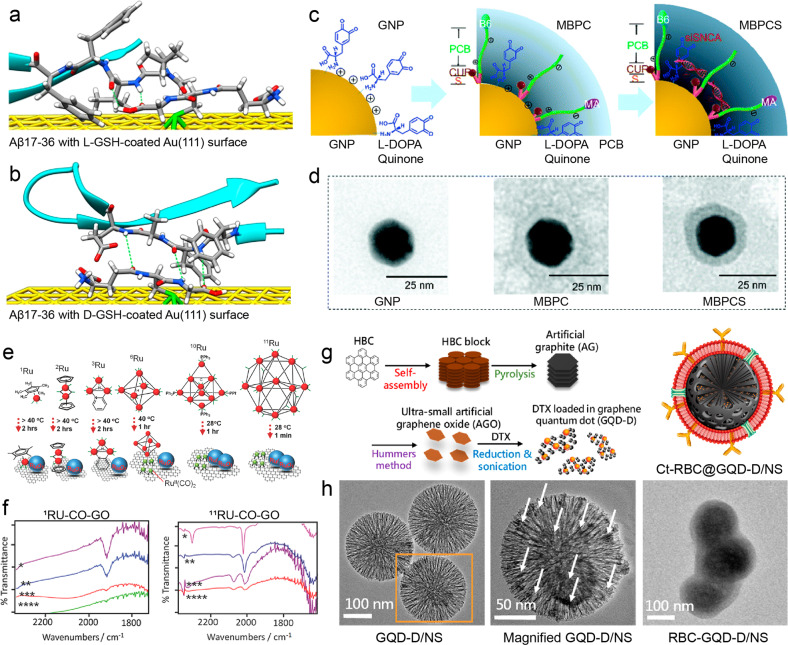
Gold nanomaterials and carbon materials for brain-targeted drug delivery. **a** Simulated structures of Aβ17-36 with L- and **b** D-GSH-coated Au (111) surface obtained from molecular docking simulation. Reproduced with permission. Copyright 2020, Nature Publishing Group. **c** Schematic illustration of synthesis and **d** TEM images of mazindol-B6 peptide-PCB-S-curcumin-siRNA (MBPCS) and intermediate products. Reproduced with permission. Copyright 2019, Royal Society of Chemistry. **e** Structures and reactivity of the various nuclearities of Ru-CO clusters on GO, and **f** FTIR spectra of 1Ru-and 11Ru-CO. Reproduced with permission. Copyright 2018, Wiley-VCH. **g** Schematic illustration of synthesis of drug-loaded graphene quantum dots (GQD-D) and Cetuximab-labeled red blood cell membrane-coated GQD-D anchored inside the nanosponge (Ct-RBC@GQD-D/NS) and **h** their TEM images. Reproduced with permission. Copyright 2019, American Chemical Society

More recently, chiral gold nanoparticles have received attention in both nanotechnology and biomedicine.^292,293^ Tang and co-workers firstly revealed that the BBB penetration of gold nanoparticles is clearly affected by their chirality (Fig. 7a, b).^294^ It is known that GSH transporters at high level in the brain promoted the BBB permeability of GSH-capped nanoparticles. In this work, the authors indicated that the D-GSH-stabilized gold nanoparticles at 3.3 nm (D3.3) possessed higher brain distribution compared to its enantiomer (L-GSH-stabilized nanoparticles, L3.3). Together with a larger binding affinity towards amyloid-β of D3.3, the introduction of chiral ligands endowed the nanoparticles with improved rescue of behavioral impairments in Alzheimer’s disease. This study undoubtfully provides a deeper understanding of nanotechnology-enabled BBB crossing by ligand functionalization.

Gold nanoparticle-based drug delivery systems in unconventional structure other than sphere particle have been designed for enhanced brain-targeted delivery. For example, a switchable nanoplatform for co-delivery of gene and chemical drugs was nano-fabricated by Zhang and his team (Fig. 7c, d).^295^ The modification of nanoparticles with B6 peptide, mazindol, β-thiother ester bonded-copolymer endowed the nanosystem with brain targeting ability, high affinity towards dopaminergic neurons, and ROS-response drug release, respectively. It significantly improved the targeted delivery of curcumin and small interfering RNA to accomplish a synergistic delivery overcoming the issue of Parkinson’s disease. Interestingly, the levodopa attached on the gold surface provides a Fe^3+^-enabled assembly in neurons for enhanced computed tomography. Microenvironment-mediated switching between self-assembly and disassembly by gold nanoparticles was developed for brain tumor treatment. The endogenous factors in brain tumor such as low pH and higher GSH could triggered the transition of assembly–disassembly state to improve theranostic efficacy.^296,297^ Moreover, hybrid nanoparticles that are composed of gold and other materials inherited the intrinsic properties of each component. Bimetallic nanoparticles (gold and palladium) modified with quercetin could serve as a potential inducer for the therapy of Alzheimer’s disease.^298^

Currently, some gold nanomaterials-based systems are undergoing pre-clinical and clinical trials. The limitations and strengths of these systems should be further investigated and summarized, provide a guidebook for the community. Comparative studies are required to explore the effects of shape, size, surface charge, and other factors on nano-bio interactions. In addition to the BBB crossing, research on the biodistribution and elimination of gold nanomaterials in the brain should be continued.

### Carbon materials

Carbon materials including carbon dots (CDs), carbon nanotubes (CNTs), graphene, and graphene oxide have been considered as promising agents for biomedical applications.^299–301^ They are evolving as functional materials not only for drug delivery but also, in parallel, for imaging, diagnosis and other treatments while proving negligible side effects.^302–305^

CDs have been exploited as biocompatible nanocarriers for brain-targeted drug delivery due to their tunable properties, photostability, small size, and facial synthesis.^306^ Leblanc et al. successively reported the development of two CDs-based nanocarriers to treat glioblastoma brain tumor and Alzheimer’s disease.^307,308^ The CDs for brain tumor treatment were conjugated with transferrin, epirubicin, temozolomide via EDC/NHS reactions with carboxylic groups on CDs surface. And the CDs for Alzheimer’s disease treatment were fabricated by an ultrasonication-mediated strategy, producing amphiphilic yellow-emissive particles at a size of 3 nm. Such CDs with amphiphilicity could cross the BBB through passive fusion.

Graphene oxide (GO) and reduced GO (rGO) are two representative graphene-based materials.^309^ They were well known for their electro-mobility, and photothermal properties, high specific surface area, giving them great potentials in targeted drug delivery. Drug molecules could be conveniently loaded into graphene-based delivery system via non-covalent interactions (π-π stacking, hydrophobic interactions, hydrogen bonding, and electrostatic interactions).^310^ Further, the -OH and -COOH groups of GO and rGO provides a feasibility to conjugate with targeted molecules and material matrix. Interestingly, the graphene-based materials themselves have the potential therapeutic effects in treatment of CNS disorders. They have been used to inhibit misfolding and aggregation of amyloid-β protein. By loading dauricine, a dibenzyl tetrahydroisoquinoline alkaloid that alleviates brain inflammation, GO sheets were verified to possess the therapeutic effects for Alzheimer’s disease.^311^ In addition to small-molecule drugs, clusters such as Ru^II^(CO)_2_ species could be absorbed onto GO (Fig. 7e, f).^312^ Under photothermal activation, carbon dioxide was released for the treatment of vascular diseases, like stroke remediation. Obviously, conjugation of functional ligands has been explored to facilitate the BBB transcytosis of graphene-based materials, such as lactoferrin, porphyrin, and transferrin.^313–316^ Physico-chemical properties such as size and surface charge also have impact on BBB penetration efficiency of GO and rGO.^309^ Graphene quantum dots at a size range from 2 to 4 nm have higher BBB permeability than GO at a size from 5 to 20 nm, indicating the crucial role of particle size in brain drug delivery.^317^ Due to the small size, graphene quantum dots could be encapsulated onto mesoporous nanostructure and mediate the theranostic penetrative delivery of drug and photolytics (Fig. 7g, h).^318^ her engineering of cell membrane onto the hybrid particles prolongated the blood circulation, leading to enhanced accumulation at brain tumors.

CNTs can been divided into two types, namely single-walled CNTs (SWCNTs) and multi-walled CNTs (MWCNTs) depending on the inner graphene layers. Unique surface chemistry and walled structure of CNTs permit drug loading and polymer conjugation for targeted delivery to brains.^319^ The CNTs for drug delivery were usually modified with functional groups such as -NH_2_ or -COOH, providing reactive sites. Raza developed PEGylated CNTs after -NH_2_ modification for delivery of mangiferin, a potential anti-cancer drug as a nanomedicine for brain tumor.^320^ Carboxylated SWCNTs were utilized for brain delivery of levodopa for the treatment of Parkinson’s disease.^321^ Chen et al. combined a mixture of brain targeting peptide, glioma targeting and polyethyleneimine with carboxylated MWCNTs to form nanomedicine for orthotopic brain tumors.^322^ The therapeutic agents could be loaded within the systems via either covalent conjugation or non-covalent bonding. However, it remains public concern for human health as exposure of CNTs may cause cytotoxicity in CNS,^323,324^ and more toxicological profiles are needed before they can be applied in clinical trials.

### Iron oxide nanoparticles

Iron oxide nanoparticles (IONPs) are a class of widely used nanomaterials for biomedical applications including drug delivery, bioimaging, hyperthermia therapy, and magnetic separation.^325–327^ Magnetite (Fe_3_O_4_), hematite (α-Fe_2_O_3_) and maghemite (γ-Fe_2_O_3_) are three common IONPs, in which Fe_3_O_4_ nanoparticles received great interests. Of importance, customized IONPs with different size, shape, morphology, and surface chemistry could be synthesized in a controlled manner. Typically, administration of unmodified IONPs does not permit brain-targeted delivery. Their accumulation pattern in organs of rodent models was revealed as follows: spleen > blood > liver > kidney > lungs > heart > testis > brain.^328^ Covalent conjugation of targeting ligands, and/or polymeric materials such as PEG chains, carboxymethyl cellulose, dextran could distinctively facilitate the transport of IONPs across the BBB.^329–331^

**Fig. 8:**
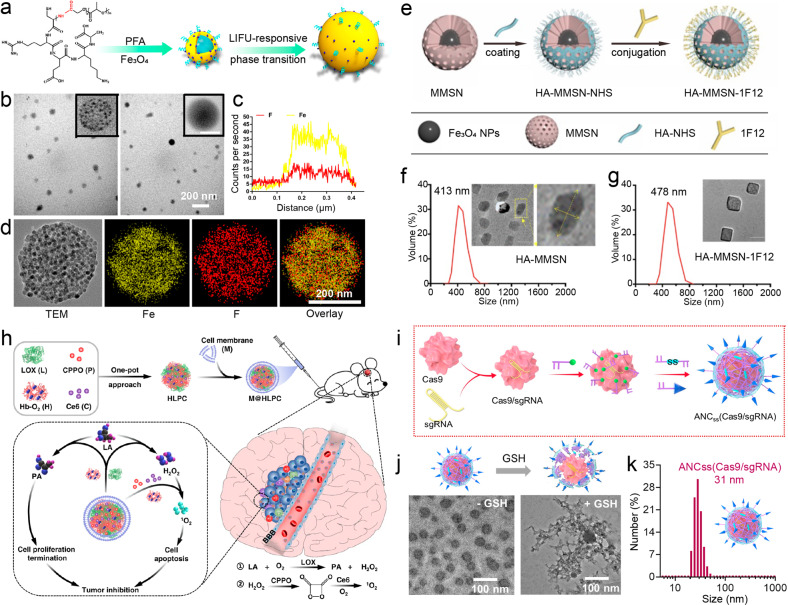
Iron oxide nanoparticles, silica nanomaterials, biomimetic nanomaterials and Cas9/RNA nanoparticles for brain-targeted drug delivery. **a** Schematic illustration of synthesis of Fe_3_O_4_ nanoparticles-PLGA-perfluorohexane (PFH) nanoparticles with CREKA peptides and **b**, **c** representative TEM images of Fe_3_O_4_-PLGA-PFH-CREKA and PLGA-PFH-CREKA nanoparticles, and **d** their elemental mapping results. Reproduced with permission. Copyright 2019, American Chemical Society. **e** Schematic illustration of synthesis of HA-MMSN-1F12 nanoparticles and **f** particle size and **g** TEM images of HA-MMSN and HA-MMSN-1F12 nanoparticles. Reproduced with permission. Copyright 2022, Ivyspring International Publisher. **h** Schematic illustration of synthesis of cell membrane-coated nanostructures and its targeted synergistic therapy of glioblastoma. Reproduced with permission. Copyright 2022, Nature Publishing Group. **i** Schematic illustration of synthesis of CRISPR-Cas9 nanocapsules and **j** their TEM images and **k** size distribution. Reproduced with permission. Copyright 2022, American Association for the Advancement of Science

Spherical IONPs or SPIONs could serve as carriers for brain-targeted delivery. For instance, IONPs synthesized by a mixture of L-aspartic acid, FeCl_3_·6H_2_O, and FeCl_2_·4H_2_O could be functionalized with carboxylic groups, paclitaxel, PEG polymer chain, and GSH step by step.^332^ The as-synthesized particles were proved to pass through the BBB with shuttle peptide and deliver the drug molecules into brain. Ni and co-authors fabricated brain-targeted IONPs for co-delivery of GSH peroxidase 4 and cisplatin for gene-chemotherapy.^333^ The small interfering RNA and the chemical drugs were loaded via EDC/NHS reaction and direct absorption, respectively. Small size IONPs were sometimes used as therapeutic agents other than the carriers. Guo developed an ultrasound-responsive nanoparticle for thrombolysis, by assembly of PLGA and targeting peptide dual-modified nanoparticles onto perfluorohexane nanodroplet (Fig. 8a–d).^334^ In another report, SPIONs, quantum dots, cilengitide were integrated into one nanoplatform, giving liposomal formulation for imaging-guided therapy. The hydrophilic cilengitide was encapsulated in the aqueous core, whereas SPIONs and quantum dots were concealed inside the lipid membrane. The SPIONs and the quantum dots used in the formulation have a size of ~20 nm and ~8 nm, respectively, and the hydrodynamic diameter of the final liposome was determined to be ~100 nm. The authors claimed the size change could promote the passive targeting via leaking vasculature (7–150 nm) and meanwhile prevent homogenous leakage of SPIONs into normal tissues.^335^

Recently, IONPs with tailored structures or multi-component IONPs-based particles were developed for brain diseases. Zhou et al. provided gallic acid-coated magnetic nanoclovers for targeted delivery of nanomedicine to brain tumors.^336^ The clover-shaped nanoparticle was synthesized by a mixture of CoFe-oleate, oleic acid, and oleyl alcohol after an elongated reaction time. The authors indicated that these nanoclovers have greater heat induction efficiency than common IONPs, leading to enhanced magnetic hyperthermia-chemotherapy combination for brain tumor treatment. Paulmurugan and co-workers synthesized polyfunctional gold-iron oxide nanostars for microRNA delivery to combat against glioblastoma.^337^ The gold-iron oxide nanostars could be obtained by consecutive seed and growth steps, giving a uniform size distribution of around 34 nm. Further coating of hybrid polymer (β-cyclodextrin-chitosan) and decoration of PEG-T7 peptide enabled efficient cargo loading and brain tumor targeting.

IONPs are widely used in the clinic for diagnostic and/or prognostic applications. The development of IONPs-based drug delivery system enabled trackable delivery and imaging-guided therapy. Recent advances of magnetotherapy offer a new opportunity for combined therapy. Thus, future work on the construction of multi-functional nanoplatform, as well as the study on how to balance the interactions between each component for optimized efficacy should be conducted.

### Silica nanomaterials

Silica nanoparticles are a type of stable, low toxic, and nanostructured bioceramics contributing their superlative properties and uses to biomedicine. Mesoporous silica nanoparticles (MSNs) characterized by ordered distribution of mesopores at a pore size between 2 and 50 nm with high pore volume and surface area are ideal candidates of starting biomaterials, particularly as drug carrier.^338,339^ The presence of silanol groups at particle surface enables the production of multifunctional derivatives of MSNs system for targeted drug delivery in the treatment of brain diseases.

There are mainly two strategies to fabricate MSNs-based delivery system for BBB crossing. First, MSNs can be employed as a drug delivery system by direct drug absorption and surface ligand functionalization. Chen et al. conjugated cRGD peptide with the MSNs and simultaneously load antineoplastic DOX by mixing.^340^ They found that such system exhibited strong permeability across the BBB and then could induce cancer cells apoptosis by releasing drugs. The transport capability of MSNs at the sizes of 20, 40, and 80 nm were evaluated by the authors and were calculated to be 44.0%, 59.2%, and 38.6%, respectively, which were all higher than that of free drugs (32.8%), indicating a size-dependent penetration mechanism. Gómez-Ruiz and co-workers prepared a MSNs-based nanoplatform for delivery of a cocktail of agents (leptin and pioglitazone) to fight against amyotrophic lateral sclerosis.^341^ Leptin, a polypeptide hormone possessing neuroprotective effects, was conjugated with -NH_2_ of MSNs through EDC/NHS coupling, and pioglitazone, an anti-inflammatory agent, was loaded through adsorption. The surface area and the pore volume before and after the drug loading were determined to be 853 m^2^/g and 0.73 cm^3^/g, 512 m^2^/g and 0.47 cm^3^/g, respectively. These results demonstrated successful incorporation of both agents. Other nanoplatforms using iRGD peptide,^342^ lactoferrin,^343^ lipoic acid,^344^ Angiopep-2,^345^ lipid bilayer,^346^ as targeting ligands also demonstrated efficient drug loading and BBB crossing of MSNs-based delivery systems.

Moreover, MSNs were used as core substrates for further materials growth, or as the carrier shell coating on other functional nanostructures including those particles mentioned above. For example, a core-shell particle system was constructed by polymerization of tetraethyl orthosilicate grafting on magnetic Fe_3_O_4_ nanoparticles for both drug delivery and magnetic resonance imaging. Afterwards, the drug-loaded Fe_3_O_4_@MSNs particles were wrapped up by activated neutrophils, producing a biomimetic theranostic platform. The authors claimed the neutrophils not only have the native ability of BBB crossing, but also could act as “living” delivery system targeting inflammatory regions for maximizing the drug bioavailability.^347^ Similar magnetic core-shell particles were developed by Luo and co-workers (Fig. 8e–g).^348^ The Fe_3_O_4_@MSNs particles were modified with both amyloid-β antibody (1F12) and CD44-targeting ligand (hyaluronic acid). Thus, after BBB penetration enabled by hyaluronic acid, the particles could specifically remove amyloid-β oligomers. In their work, the particles size had a hydrodynamic size ranging from 413 to 478 nm which is not an ideal particle size for BBB crossing. However, the authors successfully settled the problem by conjugation of CD44-targeting ligand with MSNs. Karathanasis demonstrated the BBB permeability of Fe_3_O_4_@MSNs modified with NH_2_-PEG polymer and fibronectin-targeting peptide CREKA. Thanks to the magnetic cores, external low-power radiofrequency field could facilitate deep penetration of drugs across blood–brain tumor interface.^349^

Unique properties of silica nanomaterials have made them ideal candidates to be used as drug carriers. In particular, the superior surface chemistry and structural feature of the materials distinctively improve the drug loading capacity. Thus, efficient and standardized production protocols of silica materials should be developed to achieve reproducibility.

### Biomimetic drug delivery systems

Engineered materials could exert their therapeutic benefits in drug delivery as an enormous quantity of nanoparticles with customized physio-chemical properties according to the requirement in disease treatment. In the past decade, camouflaged drug delivery systems that using biomimetic materials were developed to traverse BBB for brain-targeted drug delivery.^350–352^ The earliest work can be traced back to twelve years ago that Zhang’s group demonstrate an erythrocyte membrane-camouflaged polymeric nanoparticle for drug delivery.^353^ Since then, in addition to membrane-enabled technology, alternative strategies including biomacromolecule-enabled strategy,^354^ exosome-mediated carrier,^355^ virus-inspired synthesis,^356^ extracellular vesicles bionic method,^357^ and bacteria bionic strategy^358^ have been adopted in preparation of custom-build nanocarriers. In terms of the synthesis process, the biomimetic nanomaterials for brain delivery can generally be categorized into two strategies, namely bottom-up and top-down strategies.

The bottom-up strategy for particle construction mainly includes the use of a massive number of targeting ligands derived from living systems. From small molecules, short peptides, nuclei acids, to various functional proteins, researchers have created a toolbox of targeting ligands for nanoparticles decoration. In addition to direct conjugation to nanoparticles that have been mentioned in previous sections, targeting ligands are also able to assemble into the nanostructures in assistance with polymers to accomplish the goals of targeted drug delivery. A synthetic protein nanoparticle assembled by HSA, cell-penetrating peptide (iRGD), reactive macromer (OEG) and siRNA against STAT3, a key factor related to tumor progression, was engineered by Lahann.^359^ Under the treatment of electrohydrodynamic jetting, the OEG macromer polymerized and covalently interacted with the lysine residues from albumin, giving nanoparticles at an average size of 115 nm. In vitro and in vivo study evidenced the BBB crossing and tumor accumulation of the protein-mediated nanoparticles. To improve the BBB permeability of cytotoxic T-lymphocyte associated antigen 4 (a-CTLA-4) and programmed cell death-1 (a-PD-1) for immunotherapy of brain glioma, Ljubimova and co-workers described a nanoscale immunoconjugate system.^360^ The immunoconjugate was obtained by covalently attaching antibodies (a-CTLA-4 IgG2b, or a-PD-1 IgG) to poly(β-L-malic acid) polymers, with pre-conjugation of anti-mouse transferrin receptor antibody, and trileucine. Some natural cell membranes such as exosomes without any functionalization have ability to cross the BBB.^335,361^ The delivery efficiency is mainly dependent on the lipid membrane and the active proteins which mediate the internalization between exosomes and target cells. In addition, some exosomes improved the drug delivery efficiency by increasing the BBB membrane fluidity. Interestingly, some natural exosomes not only could penetrate BBB, but also accumulate at brain tumor site^362^ or inflammatory region,^363^ serving as dual targeted delivery systems.

Biomimetic nanomaterials developed with top-down cell-engineered approach inherited complexity of their source cells, bypassing cumbersome synthesis process for particle construction. Cellular internalization and cell membrane coating as typical top-down strategies endow the nanoparticles with bionic communications with biological entities including membrane fusion, cell tropism, immune evasion, cell-cell recognition, which in all contributes to targeted drug delivery. Researchers have reported a variety of biomimetic nanomedicines by engineering whole cells for synthesis of whole-cell-based systems, and parts of cells for semi-biological biomimetic systems. Zhang developed a neutrophil-mediated drug delivery system by anti-cancer drug encapsulation into cationic liposomes, followed by internalization by tumor-associated neutrophils.^364^ The camouflaged delivery system maintained the physiological features, which could efficiently traverse BBB and migrate to inflammatory sites such as tumor parenchyma. Interestingly, the internalization by leukocytes could complete in vivo. Qin et al. prepared cRGD-modified liposomes and demonstrated their enhanced uptake by monocytes and neutrophils in vivo due to the high and specific binding affinity.^365^ As known, leukocytes are promptly recruited when neuroinflammation occurs in brain disorders. This cell-mediated delivery strategy could thus facilitate the migration of drug-loaded liposomes across BBB in vivo. Thanks to the homotypic recognition of membrane components, cell membrane coating is an alternative method to fabricate biomimetic delivery system. For example, cell membrane harvested from glioma U251 cells was used as a chaperon to increase brain tumor targeting of delivery system (Fig. 8h).^366^ The self-assembly nanoparticles composed of hemoglobin, lactate oxidase, chlorin e6, and chemiluminescence reagent were co-extruded with tumor cell membranes to produce membrane-coated nanoparticles. Such system displayed efficient BBB penetration and tumor-targeting capability. In some cases, due to similar amphiphilicity between cell membrane and liposome, the functional proteins from the membranes could be inserted inside the liposomal bilayer after protein fusion, generating proteolipid nanoparticles. Zheng et al. prepared biomimetic proteolipid nanoparticles by embedding glioma cell membrane proteins into indocyanine green-loaded liposomes.^367^ Unlike membrane-coated polymeric “hard” nanoparticles such as PLGA nanoparticles, these membrane-camouflaged nanoparticles were proved to be “soft” and the active proteins were easy to be inserted. Further study on platelet-camouflaged delivery system also confirmed the enhanced drug delivery in multiple tumor models including glioma, which was enabled by platelet membrane proteins.^368^ Such delivery systems largely improved the drug accumulation in brain tumor via homologous binding mechanism. To further optimize the membrane-mediated delivery with region-specific targeting, biomimetic systems could be labeled by targeting ligands via covalent or non-covalent conjugations. Ren and co-authors modified the exosome-based system with LDL peptide by simply co-incubation.^369^ The high binding affinity of the peptide and LDL receptor promoted the BBB penetration, glioma distribution and cellular uptake. Similar strategy was performed in a delivery system for brain tumor treatment by using conjugation of T7 peptide.^339^

### Other materials

In addition to the aforementioned nanomaterials, development of biotechnology and materials engineering offers other opportunities for BBB crossing. Black phosphorus (BP) is two-dimensional layered semiconducting material with high drug loading capacity, efficient photothermal conversion, and satisfactory biocompatibility.^370^ Chen prepared a BP-based delivery system consisted of brain targeting peptide lactoferrin and Paeoniflorin-loaded BP nanosheets.^371^ Elevated BBB permeability of the system was observed due to a combination of lactoferrin-mediated BBB transcytosis and photothermal effects. Some examples of metal or metal oxide-based materials (e.g., MnO_2_, MgO, TiO_2_) have been exploiting in carrier construction by taking advantages of their catalytic effects and imaging functions.^372–375^ More recently, variants of the AAV have been investigated for its potential use for BBB penetration. Nanocapsules containing single CRISPR-Cas9 and GSH-sensitive polymeric shell for glioblastoma gene therapy were designed by Shi and co-workers (Fig. 8i–k).^376^ The particle core of Cas9/sgRNA complexes were decorated with positively charged polymer via electrostatic interactions, as the particle size increased from 17 nm for naked Cas9/sgRNA to 31 nm.

## Conclusion and perspectives

BBB is a natural barrier protecting the brain from the entrance of toxins and pathogens. However, intact BBB consisted of endothelial cells and tight junctions impedes the brain permeability of therapeutic agents, which largely comprises their therapeutic efficacy of CNS diseases. Along with the rapid development of materials science and nanotechnology, various strategies for BBB regulation and crossing have been developed and engineered delivery systems with unique physio-chemical properties and multifunctional motifs were prepared for enhanced drug delivery via different BBB crossing pathways. This review introduced the basic information of BBB structure and physiology and discussed the different strategies to enhance BBB crossing in detail as well as the bypassing routes and mechanisms. Recent progress of various types of drug delivery systems and their distinguishing attributes for BBB crossing were summarized. The engineered drug delivery systems with appropriate physio-chemical properties, multi-functional modules, and good biocompatibility guaranteed excellent performance of BBB penetration for enhanced drug delivery.

Although extensive achievements have been made in this community with several FDA-approved trials, there is still a long way to go from clinical popularization and translational application. More efforts on both development of materials engineering and biomedicine are needed to bridge the gap. We list some pointel aspects for future research as following: (1) More appropriate in vitro and in vivo models for evaluation of BBB permeability. Currently, cell monolayer model (e.g., Transwell model) was widely used to verify the BBB crossing and calculate the penetration efficiency. Yet, a compliant three-dimensional BBB model is more preferred to investigate the role of blood flow and drug transport in BBB maintenance. Recent advances in 3D printing technology may provide an alternative approach to in vitro BBB study;^377,378^ (2) Targeted delivery with better spatial and temporal precision. Although abovementioned strategies provide means to improve brain targeting, none of them could complete brain region-specific delivery, which is crucial for treatment of brain disorders. Strategy of targeting ligand functionalization significantly relies on the receptor expression, and stimuli-triggered BBB disruption has limited resolution. Drug release in a time-controlled manner would also be highly favored. Treatment of paroxysmal disorders such as epilepsy required timely drug release. (3) Safety issues should be addressed before clinical translation. In addition to conventional acute and chronic toxicity evaluation in cellular, organ, tissue, and system levels, studies on distribution and metabolic fate of nanomaterials in a long-term period should be conducted. Even though most literature claimed satisfactory biocompatibility in their reports, toxicological studies on organic and inorganic drug delivery systems revealed neurotoxicity and inflammatory damages in the brain.^379–381^ Researches on integrity and function of BBB at molecular level after non-invasive regulation should also be performed to verify the biosafety. (4) The interactions between drug delivery systems, cargo drug molecules, and cells should be identified. A deeper understanding of complex interactions and computational modeling, including machine learning algorithms,^382^ could provide complementary insight on empirical design of drug delivery system. (5) Scalability and reproducibility should be guaranteed. New methodologies for carrier construction and drug loading will facilitate their practical uses. (6) In addition to drug delivery, a combinational delivery of other agents such as imaging probes and biosensors is anticipated to improve the therapeutic efficacy by fabricating theranostic nanoplatforms. Targeted drug delivery is a multi-disciplinary study involving biotechnology, chemistry, material science and medicine. Considered the promising results that shown in this field, we envision that further continuous interdisciplinary cooperation will build a broader platform for enhanced BBB crossing for the treatment of brain diseases in the future.
